# Nursing Image Among Pre‐Nursing Students: A Mixed‐Methods Systematic Review with Future Directions on Recruitment Strategies

**DOI:** 10.1111/inr.70234

**Published:** 2026-09-25

**Authors:** Shin Syuen Lee, Joko Gunawan, Siriwan Lim, Ivan Rubbi, Vivien K. G. Lim, Sok Ying Liaw

**Affiliations:** ^1^ Alice Lee Centre for Nursing Studies Yong Loo Lin School of Medicine National University of Singapore Singapore; ^2^ School of Nursing Ravenna Campus University of Bologna Bologna Italy; ^3^ Alice Lee Centre for Nursing Studies Yong Loo Lin School of Medicine Department of Management & Organization NUS Business School National University of Singapore Singapore

## Abstract

**Aim:**

To synthesize existing evidence to inform targeted recruitment strategies and interventions.

**Background:**

The ongoing global shortage of nurses presents a significant threat to healthcare delivery. Addressing this requires an understanding of why pre‐nursing students choose to or avoid nursing as a career. A key deterrent is the profession's negative image, often associated with “dirty work.” However, current literature lacks a comprehensive view of the factors shaping these perceptions.

**Methods:**

Nine databases were searched from inception to October 2024. Eligible studies included quantitative, qualitative, and mixed‐methods designs focusing on pre‐nursing students’ perceptions of nursing image. A five‐stage framework analysis structured the findings, and Bronfenbrenner's social–ecological model categorized factors at individual, interpersonal, and societal levels. The JBI Convergent Integrated Method integrated qualitative findings and qualitized quantitative evidence according to these levels.

**Results:**

Thirty‐two studies (9034 participants) from 14 countries were included. Individual‐level factors included gender stereotypes, knowledge of the profession, perceived work attributes, and intrinsic motivation. Interpersonal‐level factors included the role of family, peers, and exposure to nursing professionals. Societal‐level factors included media portrayals, sociocultural norms, and targeted recruitment interventions.

**Conclusion:**

Factors converge across levels, and passive visibility alone is insufficient to shift entrenched views. Post‐COVID “hero” narratives may increase visibility but also reinforce associations with fatigue and difficult working conditions. Improving pre‐nursing students’ perceptions of nursing image requires sustained, coordinated, multilevel recruitment strategies.

**Implications for nursing:**

Educators and healthcare institutions may consider strategies that challenge gender stereotypes, engage parents and peers, provide structured exposure to nursing roles, and strengthen leadership‐focused media portrayals.

**Implications for health policy:**

Recruitment strategies should be supported by policy and aligned with efforts to address structural conditions that shape nursing image, with the ultimate goal of strengthening the future nursing workforce and as part of workforce planning.

## 1 Introduction

Globally, nursing represents the backbone of healthcare systems, with over 29 million nurses in the global health workforce (World Health Organization [WHO] 2024). Despite their critical contributions, the profession faces significant challenges, including a projected global deficit of 4.8 million nurses by 2030 and persistent shortages in South‐East Asia and the WHO Eastern Mediterranean Region (Boniol et al. 2022). These shortages are exacerbated by perceptions of nursing image that often undermine its professional status and intellectual rigor (Chen et al. 2020; Liaw et al. 2016; Toth et al. 1998; Woods‐Giscombe et al. 2020), perpetuating outdated stereotypes that frame nursing as a caregiving, subordinate, and female‐dominated profession (Teresa‐Morales et al. 2022).

A positive nursing image plays an important role in shaping career choices. The concept of nursing image encompasses public perceptions, professional identity, media representation, and collective views of healthcare professionals (Rezaei‐Adaryani et al. 2012). The International Council of Nurses [ICN] (2021) defines nursing as an autonomous and collaborative care profession that integrates health promotion, illness prevention, and care for the ill and end‐of‐life support. Attitudes toward nursing refer to how individuals view nurses’ roles, values, responsibilities, and professional activities (Toth et al. 1998). Extant research generally focuses on issues related to manpower, such as recruitment and retention, yet nursing image has received relatively less attention from scholars. Bridging this gap is essential, as nursing image significantly influences public perceptions, affecting career interest in the profession (Elmorshedy et al. 2020; Hoeve et al. 2014). Strengthening the nursing workforce pipeline therefore requires a clearer understanding of how the nursing image is perceived.

Strengthening the nursing workforce pipeline therefore requires a clearer understanding of how nursing is perceived before individuals enter nursing education or practice. For many potential entrants, these perceptions develop during adolescence and young adulthood, pivotal life stages where youth develop attitudes and beliefs about the world of work and professions. During these formative years, adolescents are particularly susceptible to social influences shaping their career decisions and perceptions (Ciranka and Van den Bos 2019). Research suggests that positive perceptions of nursing image may be associated with greater interest in pursuing nursing as a career (Hadid and Khatib 2015; López‐Verdugo et al. 2021). Therefore, addressing misconceptions and fostering a positive nursing image may support efforts to attract potential entrants to the profession.

Recent systematic reviews have approached nursing image and career decision‐making from different perspectives. These reviews drew on populations spanning multiple stages of professional entry, from practicing nurses and healthcare professionals to undergraduates already enrolled in health‐profession programs and unrestricted general‐public samples, without isolating individuals prior to nursing entry (Glerean et al. 2017; López‐Verdugo et al. 2021; Rezaei‐Adaryani et al. 2012; Wu et al. 2015). Glerean et al. (2017) identified misconceptions about nursing as a career among young people, including misunderstandings about autonomy, working conditions, and career pathways. López‐Verdugo et al. (2021) found stereotypes and general‐public misconceptions that undermine trust and professional recognition. Rezaei‐Adaryani et al. (2012) found antecedents such as media portrayals and professional behaviors affecting the general view of the nursing image, lacking focus on specific populations. Other reviews focused more directly on nursing career choice. Wu et al. (2015) examined factors influencing healthcare undergraduates’ career decisions, noting key deterrents such as perceived low financial rewards. This population included individuals already enrolled in nursing and other health‐profession programs, rather than those yet to select a health career. Gunawan et al. (2025) synthesized global evidence on factors influencing nursing students’ decision to pursue a nursing career. While these reviews provide valuable insights, they do not offer a synthesis that is restricted to individuals before entry into nursing education or practice, and their focus on nursing as a career rather than nursing image leaves gaps in understanding the broader dimensions and influencing factors.

Existing reviews also reveal methodological limitations, including inconsistent definitions of nursing image (Rezaei‐Adaryani et al. 2012; Wu et al. 2015), the conflation of nursing image with nursing career outcomes (Wu et al. 2015), limited database searches (Glerean et al. 2017), and insufficient focus on populations before entry into nursing education or practice (López‐Verdugo et al. 2021). Moreover, the pandemic has brought nurses to the forefront of public attention, with media publicity hailing them as ‘heroes’ for their selfless actions (Morin and Baptiste 2020). Hadid and Khatib (2015) further highlighted that heroism became embedded within a militaristic narrative, positioning nurses as soldiers engaged in a metaphorical battle against illness. Media companies such as Marvel Comics collaborated with healthcare organizations by showcasing nurses’ clinical expertise and frontline presence in managing the COVID‐19 pandemic (Marvel 2020). However, while the COVID‐19 pandemic elevated the visibility of nurses as frontline healthcare professionals, its impact on pre‐nursing students’ perceptions of nursing image remains underexplored.

To our knowledge, there is no existing review article focusing on pre‐nursing students’ perceptions of nursing image. Given these gaps in the literature, this mixed‐methods systematic review aims to systematically synthesize evidence on factors influencing pre‐nursing students' perceptions of nursing image, with the research question as follows:

What are the factors influencing pre‐nursing students’ perceptions of nursing image?

## 2 Methods

### 2.1 Protocol Registration

The review protocol was registered in the International Prospective Register of Systematic Reviews (PROSPERO; CRD420250628497) on March 24, 2025. Registration occurred after completion of the literature search and commencement of study screening, but before data extraction, quality appraisal, and data synthesis.

### 2.2 Search Strategy

We conducted a preliminary search on PROSPERO and Google Scholar to avoid duplicating systematic reviews or meta‐analyses. We then implemented a three‐step comprehensive search strategy from inception to October 2024. First, we systematically searched nine databases using index and key terms (Supplementary Table S1) that were developed in consultation with an experienced librarian team and based on the methodology outlined in the Cochrane Handbook (Higgins et al. 2023). The databases included PubMed (via NIH), Cumulative Index to Nursing and Allied Health Literature (CINAHL via EBSCOhost), Education Resources Information Center (ERIC via ProQuest), Medline (via Ovid), Excerpta Medica Database (EMBASE), ProQuest Dissertations and Theses, Web of Science, Scopus, and PsycINFO. Second, author 1 (LSS) searched clinical trial registries for ongoing trials. Third, we screened reference lists, specialized journals, and gray literature for potential trials. We used Endnote 21 software (Clarivate Analytics 2013) to manage references and remove duplicates.

### 2.3 Eligibility Criteria

Supplementary Table S2 outlined the eligibility criteria. We included studies that explored pre‐nursing students’ perceptions of the nursing image using quantitative (e.g., cross‐sectional, pre–post intervention studies, RCTs), qualitative, or mixed‐methods designs. In this study, pre‐nursing students are operationally defined as individuals who had not commenced nursing education or entered nursing practice at the time of data collection. As perceptions of nursing may also influence individuals outside formal schooling who remain potential entrants to the profession, the eligibility criteria additionally included a small number of other pre‐nursing populations. These included individuals in transitional educational stages (UNESCO Institute for Statistics 2012), school leavers, young adults at the career‐selection stage, pre‐nursing applicants, and non‐nursing university or community participants for whom nursing remained a potential educational or career pathway. Eligibility was therefore determined by participants’ position before entry into nursing education or practice rather than by a fixed age or current student status alone.

In this review, nursing image was operationalized as students’ perceptions of nursing as a profession, including perceived roles, professional status, stereotypes, values, and work‐related attributes. Career intentions, preferences, or choices were not treated as nursing image outcomes. Systematic reviews were the only study type excluded. Given that educational exposure can significantly shape perceptions of nursing image (Ten Hoeve et al. 2017), studies focused on nursing students, practicing nurses, or other healthcare professionals were not eligible.

The outcome of interest was the identification of factors influencing pre‐nursing students’ perceptions of nursing image, as measured by validated instruments or interview findings. Career‐related variables were considered only when they reflected underlying perceptions of nursing image and were not synthesized as behavioral outcomes. Career preference, behavioral intention, application, enrolment and recruitment were treated as conceptually distinct career‐related outcomes. Such outcomes were reported only to contextualize their relationship with nursing image and were not interpreted as nursing‐image measures. To provide a comprehensive understanding of how these perceptions may have evolved over time, studies from any publication year were included. Both published and unpublished sources in English and Mandarin were considered, while studies published in other languages were not eligible.

### 2.4 Data Extraction

Following the Preferred Reporting Items for Systematic Reviews and Meta‐Analyses (PRISMA) checklist guidelines, author 1 (LSS) developed a structured data extraction form and extracted the following information: author(s), year of publication and country, study design, population characteristics and sample size, study aim, data source and instrument, whether the study was theory‐based, key review‐relevant findings, and the main factors identified.

We piloted the data extraction form using a sample of three to six studies to ensure the items’ accuracy and appropriateness (Higgins et al. 2023). After confirming the items’ suitability, two reviewers (LSS and JG) verified all extracted data.

### 2.5 Quality Appraisal

We used the Mixed‐Methods Appraisal Tool (MMAT) to evaluate study quality across various research designs included in this review (Hong et al. 2019). The MMAT is well‐suited for mixed‐methods systematic reviews, as it provides specific appraisal criteria tailored to different study types, including qualitative, randomized controlled trials, nonrandomized, quantitative descriptive, and mixed‐methods studies. We assessed each study based on five core criteria, with responses categorized as “yes,” “no,” or “can”t tell.‘’ Consistent with MMAT guidelines, we did not compute an aggregate quality score, which provided a more detailed presentation of each criterion.

Author 1 (LSS) independently conducted the quality assessment, and author 2 (JG) independently verified the ratings to confirm consistency. We discussed any disagreements in assessments and resolved them by consensus. We measured interrater agreement using Kappa statistics (Landis and Koch 1977). A total of 231 items were evaluated, with one discrepancy noted where the response was changed from “Can't Tell” to “No” by the second reviewer (JG). The observed agreement was 99.57%, with the expected agreement accounting for chance at 33.33%. We calculated a Kappa coefficient of 0.994, reflecting almost perfect agreement between the two reviewers (Landis and Koch 1977).

### 2.6 Data Synthesis

We synthesized the data using a mixed‐methods approach guided by the five‐stage framework analysis method (Brunton et al. 2020). This approach provided a structured way to organize and integrate findings across individual, interpersonal, and societal levels of influence on the nursing image.

After familiarizing ourselves with the data, we selected the social–ecological model (SEM) (Bronfenbrenner 1989) to categorize influencing factors. We extracted qualitative and quantitative data in parallel. We summarized the qualitative data to identify key factors shaping perceptions of nursing and extracted quantitative outcomes that reflected nursing image‐related perceptions, such as perceived roles, stereotypes, status, autonomy, and work attributes.

We then applied the Joanna Briggs Institute (JBI) Convergent Integrated Method (Stern et al. 2020) to merge qualitative and quantitative results. Quantitative nursing‐image findings were transformed into descriptive textual statements that retained the relevant population, nursing‐image construct, direction of the finding and statistical significance, where reported. These statements were compared with qualitative findings addressing the same factor within the SEM framework. Where directly comparable evidence was available, the relationship was classified as confirmation, expansion, or contradiction. Supplementary Table S3 presents illustrative examples of this integration. Career intention, preference, application, enrolment, and recruitment outcomes were retained separately and were not transformed into nursing‐image findings. Subsequently, we used narrative synthesis to describe patterns within the SEM framework.

Two authors (LSS and JG) reviewed the synthesis process and resolved any uncertainties through discussion to ensure consistency and rigor.

## 3 Results

### 3.1 Study Selection

**FIGURE 1:**
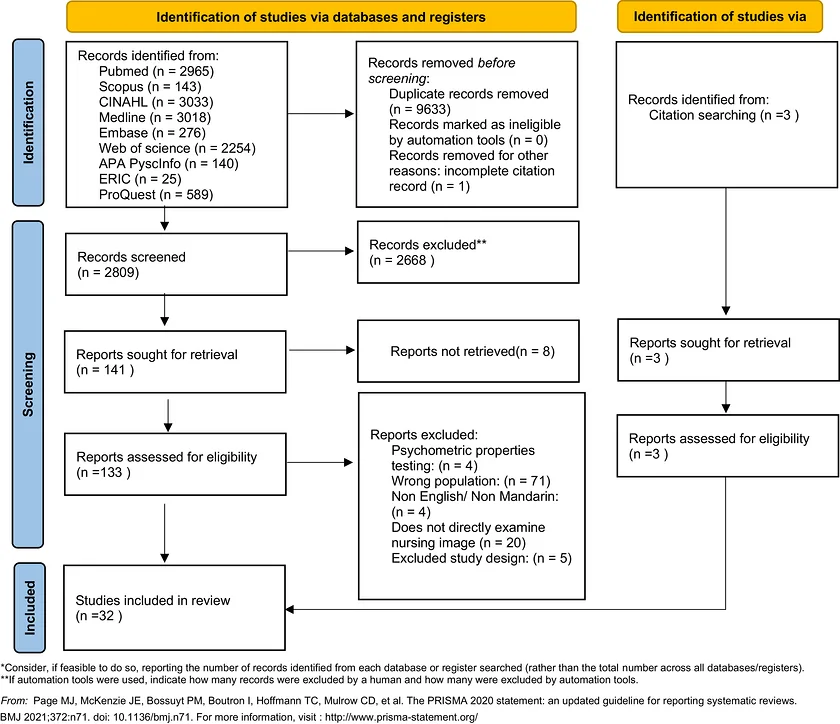
PRISMA flow diagram.

Figure 1 presents the PRISMA flow diagram documenting the study selection process. We retrieved 12,443 studies from 9 databases and identified 3 additional records through citation searching. Using EndNote 21 software, we eliminated 9633 duplicates and one incomplete citation record. Two independent reviewers (LSS and JG) first screened the titles and abstracts of 2809 records for relevance according to the established eligibility criteria. We excluded 2668 studies based on titles and abstracts and sought the full text for 141 records. Out of 141 articles, eight reports could not be retrieved, leaving 133 database‐identified reports for full‐text assessment. Both reviewers agreed to include 29 articles. Three additional reports identified through citation searching were also included, resulting in a total of 32 studies in the review. We evaluated interrater reliability by calculating Cohen's Kappa = 0.77 (Landis and Koch 1977), indicating substantial agreement between the two reviewers. We recorded the reasons for excluding 104 studies at the full‐text screening stage (Supplementary Table S4).

### 3.2 Characteristics of Included Studies

The characteristics of the 32 studies included in this mixed‐methods systematic review are summarized in Supplementary Table S5. These studies were published between 1990 and 2024 and represent diverse geographical regions, including North America (United States, *k* = 15, 46.9%), the Middle East (Israel, *k* = 3, 9.4%; Kuwait, *k* = 1, 3.1%; Saudi Arabia, *k* = 1, 3.1%; United Arab Emirates, *k* = 1, 3.1%; Bahrain, *k* = 1, 3.1%; Turkey, *k* = 1, 3.1%), Asia (Hong Kong, *k* = 2, 6.3%; South Korea, *k* = 1, 3.1%; Iran, *k* = 1, 3.1%), Europe (United Kingdom, *k* = 1, 3.1%; Italy, *k* = 1, 3.1%), South America (Brazil, *k* = 1, 3.1%), and Oceania (Australia, *k* = 3, 9.4%). The geographic distribution of included studies is summarized in Supplementary Table S6.

Most of the included studies (*k* = 26, 81.3%) employed quantitative designs: descriptive studies (*k* = 11, 34.4%) were the most common, followed by cross‐sectional studies (*k* = 10, 31.3%), comparative studies (*k* = 2, 6.3%), pretest–posttest intervention studies (*k* = 2, 6.3%), and a cluster randomized controlled trial (*k* = 1, 3.1%). Sample sizes in these quantitative studies ranged from 20 to 820 participants, with an age range of 9 to 30 years. The participants were primarily high school students (*k* = 21, 65.7%), followed by middle school students (*k* = 3, 9.4%), and other pre‐nursing populations (*k* = 2, 6.3%). As most included studies focused on high school students, the findings primarily reflect perceptions formed during mid to late adolescence (WHO 2016), with fewer studies capturing earlier developmental stages such as middle school. Three studies (*k* = 3, 9.4%) utilized the Nursing Image Scale, a validated tool designed to assess perceptions of the nursing profession; three studies (*k* = 3, 9.4%) employed the Attitudes, Values, and Beliefs Questionnaire. Other quantitative studies used various researcher‐developed or adapted tools to examine nursing image. Most studies did not report socioeconomic status (SES), race or ethnicity, or school type in a consistent format. Where intersectional data were available, some patterns emerged: Öncü et al. (2022) found that female students from lower‐income backgrounds viewed nursing more positively as stable and care‐oriented work, whereas higher‐income male students regarded it as low‐status and gender‐segregated. Reiskin and Haussler (1994) similarly found that African American and Latino female students held more favorable perceptions of nursing than Asian female students. These patterns suggest that SES and ethnicity may interact with gender in shaping nursing image, though the limited and inconsistent reporting of such data across studies precludes systematic synthesis. Statistical comparisons and effect sizes from studies that reported inferential analyses are summarized in Supplementary Table S7.

Qualitative studies comprised a smaller proportion of the review (*k* = 3, 9.4%), employing semi‐structured interviews (*k* = 2, 6.3%) and focus group discussions (*k* = 1, 3.1%). These studies had smaller sample sizes, ranging from 19 to 74 participants, with an age range of 10 to 20 years. Mixed‐methods designs were used in three studies (*k* = 3, 9.4%), integrating quantitative surveys with qualitative approaches. These included focus group discussions (*k* = 2, 6.3%) and semi‐structured interviews (*k* = 1, 3.1%) to combine descriptive statistics for closed‐ended survey questions and content analysis for open‐ended responses. These studies had sample sizes ranging from 71 to 685 participants, with an age range of 15 to 20 years.

### 3.3 Quality Assessment

All included studies (*k* = 32, 100%) had clear research questions and collected data relevant to addressing these questions, providing a solid foundation for confidence in the overall synthesis.

For qualitative studies (*k* = 3, 9.4%), 100% reported appropriate alignment between data sources, data collection, and interpretation, though minor sources of bias included limited use of direct quotes to support themes. The quantitative randomized controlled trial (*k* = 1, 3.1%) demonstrated randomization and reliable outcome measures, but unblinded assessors posed a potential bias and contributed to moderate confidence in the findings. In quantitative nonrandomized studies (*k* = 4, 12.5%), sources of bias included insufficient consideration of confounding factors in study design and analysis. This may have compromised internal validity and reduced confidence in the results, which were therefore interpreted with caution. Quantitative descriptive studies (*k* = 21, 65.6%) were primarily limited by lack of representativeness of the target population and a moderate risk of nonresponse bias, which limited confidence in the results. Lastly, mixed‐methods studies (*k* = 3, 9.4%) generally demonstrated coherence in integrating qualitative and quantitative components, strengthening confidence in findings supported by convergent evidence, although some inconsistencies between methods were noted. Supplementary Table S8 illustrated the full quality assessment stratified by key domains across these studies.

## 4 Interpretative Overview: Narrative Synthesis

**FIGURE 2:**
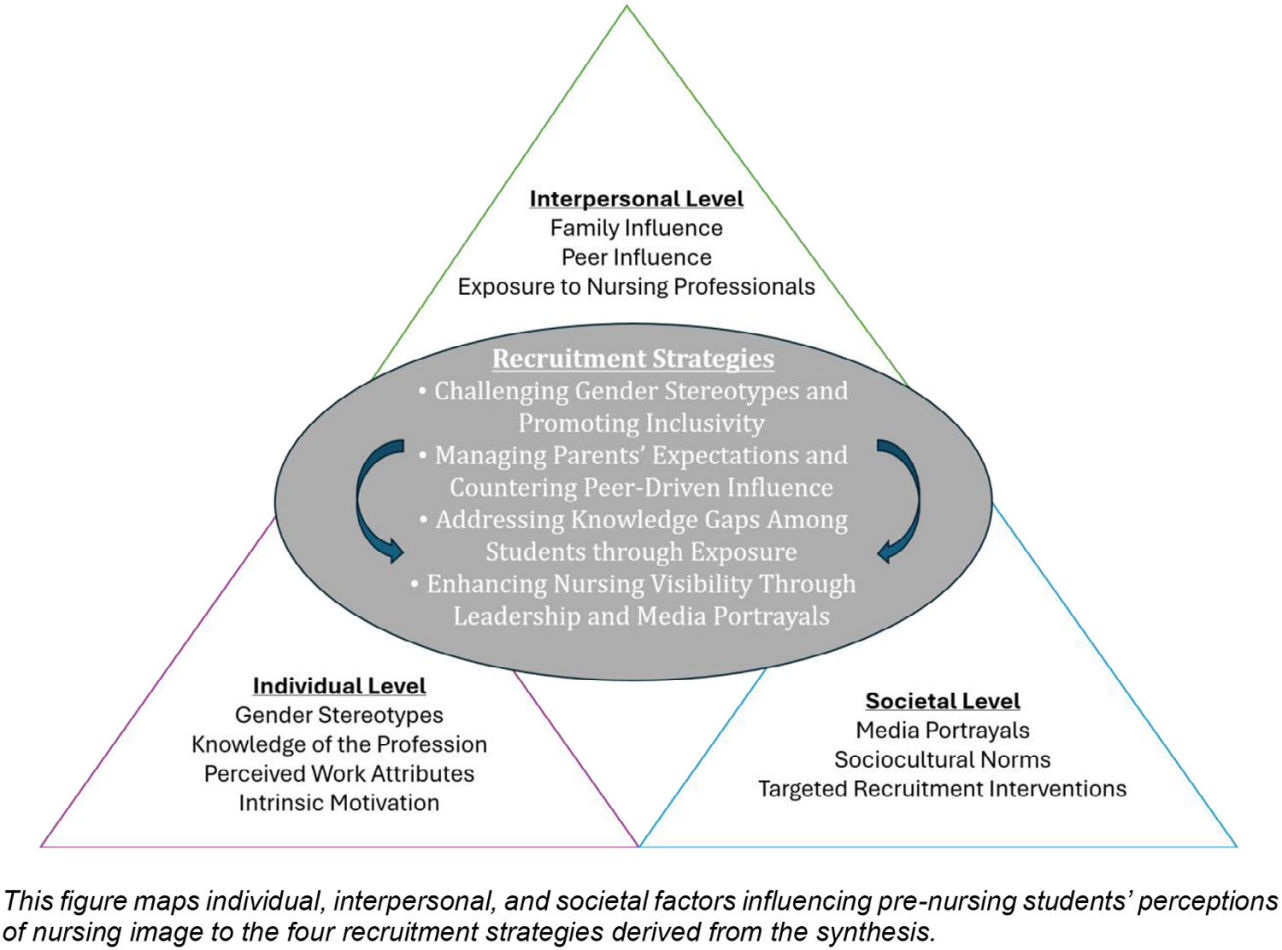
Integration of factors and recruitment strategies.

This mixed‐methods systematic review identified a range of factors influencing pre‐nursing students’ perceptions of nursing image, analyzed at the individual, interpersonal, and societal levels using Bronfenbrenner's SEM. Integration was undertaken where directly comparable qualitative and quantitative nursing‐image evidence was available. The integrated findings were predominantly confirmatory or expansive, with qualitative evidence adding context and explanation to the quantitative patterns. No direct contradiction was identified for comparable factors and populations. Supplementary Table S3 presents illustrative examples of these relationships. At the individual level, key factors included gender stereotypes, knowledge of the profession, perceived work attributes, and intrinsic motivation. The interpersonal‐level factors focused on the influence of family, peer, and exposure to nursing professionals. At the societal level, media portrayals, sociocultural norms, and targeted recruitment interventions emerged as factors influencing pre‐nursing students’ perceptions of nursing image. Figure 2 provides an overarching conceptual map that links these factors to the four recruitment strategies derived from the synthesis. It illustrates that no single intervention is sufficient on its own. Effective change therefore requires coordinated, multilayered strategies, for example, by combining student‐focused interventions that address misconceptions with family‐ and peer‐focused approaches, with broader media and policy initiatives. The strategies are discussed in detail in the Discussion section.

### 4.1 Individual Level

#### 4.1.1 Gender Stereotypes

Twelve studies indicated that gender played a significant role in shaping students’ perceptions of nursing. Male students commonly perceived nursing as a predominantly female profession, involving caregiving and supporting roles. These gendered assumptions contributed to a less favorable image of nursing among male students (Andrews 2005; Barkley and Kohler 1992). One male participant stated: “They'd think I was gay. It's a girl's job” (Hemsley‐Brown and Foskett 1999). Male high school students viewed nursing as incongruent with masculine identity, reinforcing a gendered image of the profession. Zamanzadeh et al. (2023) examined these gender differences and found that female students frequently associated nursing with caregiving, while male students found it intellectually limiting.

#### 4.1.2 Knowledge of the Profession

Eighteen articles discussed the impact of knowledge of the profession as a critical factor in shaping nursing image perceived by pre‐nursing students. Students often understood nursing as limited to bedside care, overlooking its technical and leadership aspects. Tawash and Cowman (2018) examined students’ understanding of nursing and reported significant misconceptions, with many believing that nurses played subservient roles and merely followed doctors’ orders. This was evident when one participant shared: “The workplace of a nurse is in the hospitals and clinics in which they take care of the patients according to the doctor's instructions” (Zamanzadeh et al. 2023).

#### 4.1.3 Perceived Work Attributes

Eleven studies noted that perceived work attributes also shaped students’ perceptions of nursing image. While nursing was often seen as a stable and secure career (Raymond et al. 2018), students also evaluated nursing in relation to perceived earning potential, autonomy, and social recognition (Cohen et al. 2004). Perceived limited career advancement was often associated with negative perceptions of nursing image (Hemsley‐Brown and Foskett 1999; Maymoun and Sohail 2020). Hemsley‐Brown and Foskett (1999) found that students who valued financial security viewed nursing favorably, while others perceived it as lacking prestige and long‐term growth opportunities.

#### 4.1.4 Intrinsic Motivation

Intrinsic motivation was another important factor influencing pre‐nursing students’ perceptions of nursing image. Students motivated by a desire to help others and contribute to their communities were more likely to view nursing as a fulfilling profession. This was evident in the qualitative study by Zamanzadeh and colleagues (2023), where one participant stated: “I had an innate interest in caring for and helping others, and that is why I sought information in this major.” In turn, they held more positive perceptions of nursing image. Conversely, students who prioritized financial rewards or societal recognition often had more negative perceptions of nursing image (Cohen et al. 2004; Degazon et al. 2015; Kohler and Edwards 1990; Marriner‐Tomey et al. 1990; May et al. 1991).

### 4.2 Interpersonal Level

#### 4.2.1 Family Influence

At the interpersonal level, family influence emerged as a critical factor affecting pre‐nursing students’ perceptions of nursing image. Students with family members in healthcare were more likely to hold more positive perceptions of nursing image (May et al. 1991; Rubbi et al. 2019). This was evident from the significantly higher NAQ scores among students who had a family member who was a nurse compared with those who did not (108.14 vs. 105.09, *p* < 0.05) (Rubbi et al. 2019). Conversely, families who viewed nursing as lacking in status and as financially unrewarding reinforced negative perceptions of nursing image among their children (Rossiter et al. 1999). There was a lack of studies examining family structure to better understand the nuanced impact of mixed family opinions on nursing image. For example, in a qualitative study from Iran, a participant shared: “My dad is always telling me that nursing is not an easy job and doesn't have much respect here, but my aunt is a nurse, and she says it's a meaningful career” (Zamanzadeh et al. 2023). This suggests that conflicting family perspectives may play a role in shaping perceptions of nursing image, a potential area for further exploration.

#### 4.2.2 Peer Influence

Peer perceptions often served as barriers. Several studies described how boys who expressed positive perceptions of nursing image were sometimes met with ridicule, particularly in contexts dominated by traditional gender norms such as in a country like Iran. A participant mentioned: “My friends did not like it and they disdained, in terms of class and such things, they said it is low class. They even told me not to choose the nursing, or consider it as your last choice” (Zamanzadeh et al. 2023). Negative peer stereotypes perpetuated nursing image as a low‐status, subservient profession, as observed in Hong Kong and the United Kingdom (Hemsley‐Brown and Foskett 1999; Rossiter et al. 1999). For male students, peers frequently reinforced traditional standards of masculinity through remarks such as nursing being “just for girls,” and some were laughed at for expressing interest in nursing (Murray 2002; Zamanzadeh et al. 2023). These dynamics underscored how societal gender expectations intersect with peer influences to shape perceptions of nursing image.

#### 4.2.3 Exposure to Nursing Professionals

Exposure to nursing professionals was an important facilitator, as direct interactions helped students develop a more nuanced understanding of the profession (Al‐Kandari and Lew 2005; Rubbi et al. 2019; Zamanzadeh et al. 2023). Structured school programs and initiatives allowed students to observe both the caring and technical aspects of nursing. Al‐Kandari and Lew (2005) reported that personal contact with nurses improved knowledge about the functional aspects of the profession, despite cultural and gender barriers shaping perceptions negatively. Kohler and Edwards (1990) further supported this finding, showing that personal exposure allowed students to challenge stereotypes by associating nursing with leadership and critical thinking skills rather than purely supportive roles. Opportunities facilitated by programs such as orientation days positively influenced perceptions of nursing among high school students (Rubbi et al. 2019). Hospital experiences also shaped students’ perceptions, with one student sharing her experience: “While I was in the hospital, I saw how hard the nurses work, they are wonderful people” (Zamanzadeh et al. 2023). Conversely, Murray (2002) revealed that portrayals of nurses in high‐stress, busy environments often reinforced negative perceptions of nursing image, which acted as barriers to fostering a positive nursing image.

### 4.3 Societal Level

#### 4.3.1 Media Portrayals

Six studies highlighted how media influenced perceptions of nursing image among pre‐nursing students. Among these, three studies identified media as a facilitating factor, while six studies reported it as a barrier. Media as a facilitating factor was reflected in Zamanzadeh et al. (2023), where a student noted, “I changed my mind a lot when I saw the foreign movie ‘The Nurses’. In this film, they have shown the nurses as scientific and professional individuals.” Some reported media as a barrier: Foong et al. (1999) and Joung and Shin (2018) found that media often portrayed nurses in subordinate roles, affecting perceptions of autonomy and leadership potential. Qualitative data from Zamanzadeh et al. (2023) also highlighted the discrepancy between media portrayals and actual practice, depicting nurses as simply executing doctors’ orders.

Post‐COVID studies in this review reflected this mixed picture. In Turkey, increased media coverage of exhausted frontline nurses during the pandemic did not translate into a more positive overall image, and students continued to view nursing as a low‐status job with demanding conditions (Öncü et al. 2022). In Israel, young Muslim women reported that the pandemic increased the visibility and perceived social contribution of nurses. However, this did not eradicate persistent stereotypes of nursing as a low‐status, female‐dominated occupation (Ben Natan et al. 2024).

#### 4.3.2 Sociocultural Norms

Beyond individual attitudes, several studies highlighted how wider sociocultural norms sustain and legitimize these gendered images of nursing. Long‐standing gender norms defined nursing as a feminized occupation associated with caregiving and emotional service, contributing to its lower social prestige and economic reward. In Middle Eastern contexts, gender segregation policies reinforced these norms by formally regulating nurses’ interactions with patients. Female nurses were expected to care for women and male nurses for men, which shaped professional opportunities and workplace boundaries (Al‐Kandari and Lew 2005; Elmorshedy et al. 2020; Maymoun and Sohail 2020; Tawash and Cowman 2018). In Turkey, sociocultural norms continued to portray nursing as a low‐status “pink‐collar” occupation (Öncü et al. 2022). In the United States, nursing was often described as a “helping” rather than a high‐status or leadership‐oriented profession (Degazon et al. 2015; Reiskin and Haussler 1994).

#### 4.3.3 Targeted Recruitment Interventions

Targeted recruitment interventions demonstrated potential in reshaping perceptions of nursing image among pre‐nursing students. Six studies, comprising four quantitative and two mixed‐methods trials, examined the effects of interventions on nursing image. Drenkard et al. (2002) evaluated the Inova Nursing Exploration Summer Camp and reported significant pre‐post changes in students’ perceptions that nursing offered opportunities to manage large groups, teach at university, be in demand, hold executive roles, design and direct health programs, and participate in scientific research. The proportion considering nursing as an occupation increased from 70% to 90%, which was treated separately as a career‐interest outcome. Similarly, Rubbi et al. (2019) assessed a university‐led nursing orientation and found significant higher total Nursing Attitude Questionnaire scores and scores for professional role, professional values, stereotypes, professionalism, and characteristics of nurses and nursing than students who did not attend (all *p* < 0.001). Interest in choosing a nursing degree was also higher (*p* < 0.001), but represented a separate career‐interest outcome. Droes et al. (1993) reported significant treatment effects for caring, usefulness, autonomy, scientific knowledge, and total nursing‐image scores (all *p* = 0.001). Matutina (2012) reported descriptive improvements in perceptions of nursing, although the comparisons were not statistically significant.

Mixed‐methods findings further described changes in students’ knowledge and awareness of nursing. Tawash and Cowman (2018) found that students described how interactions with nursing ambassadors challenged stereotypes, presenting nursing as a profession defined by expertise and intellectual engagement rather than traditional caregiving alone. Similarly, insights from students emphasized how career days showcased nursing's diverse opportunities, fostering a more dynamic and appealing image of the profession, with one student suggesting that an effective campaign should feature “people of all different races and all different sizes and all different interests… that would educate people and also interest people” (Murray 2002). Raymond et al. (2018) further noted that holding these programs during school hours made them accessible and impactful, as students could engage directly with nursing professionals within a familiar and convenient setting. However, these findings are based on immediate post‐intervention assessments.

## 5 Discussion

This systematic review is among the first to provide a comprehensive understanding of factors influencing pre‐nursing students’ perceptions of nursing image. The review used SEM to analyze influences at individual, interpersonal, and societal levels, providing a more nuanced understanding of how persistent stereotypes, gaps in knowledge, and structural barriers converge to shape perceptions of nursing image among pre‐nursing students. These influences operate interactively across SEM levels rather than in isolation. Societal media portrayals and sociocultural norms at the macrosystem level shape peer expectations and responses within the microsystem, which subsequently reinforce students’ individual beliefs. Such cross‐level interactions create reinforcing cycles that sustain perceptions of nursing image. These findings extend prior reviews by shifting the focus from nursing career choice to nursing image before entering nursing education. Glerean et al. (2017) similarly reported that young people often viewed nursing as caring, physically demanding, low in autonomy, and poorly understood in terms of education and career pathways. Gunawan et al. (2025), however, focused on factors influencing nursing students’ decisions to pursue nursing as a career. In contrast, this review synthesizes evidence specifically on perceptions formed before entry into nursing education or practice, and shows how gender stereotypes, role misconceptions, perceived work attributes, interpersonal influences, media portrayals, sociocultural norms, and recruitment exposure shape nursing image at an earlier stage of the workforce pipeline. Drawing these findings together, Figure 2 maps how these multilevel factors inform four overarching recruitment strategies: (1) challenging gender stereotypes and promoting inclusivity, (2) managing parents’ expectations and countering peer‐driven influence, (3) addressing knowledge gaps among students through exposure, and (4) enhancing nursing visibility through leadership and media portrayals.

### 5.1 Challenging Gender Stereotypes and Promoting Inclusivity

Findings from this review demonstrate that gendered perceptions of nursing image are not confined to specific cultural or geographical contexts but represent a persistent global trend. The association between nursing and femininity reflects long‐standing historical and sociocultural influences that continue to shape students’ perceptions. Historically, the founder of modern nursing, Florence Nightingale professionalized nursing with the assertion that “every woman is a nurse,” which further associated nursing with caregiving roles (Nightingale 1992). While this elevated nursing's moral standing, it also aligned the profession with traditional feminine virtues. This framing limited perceptions of nursing as a scientific or leadership‐driven field (Benjamin and Curtis 1985).

More recent evidence indicates that these gendered expectations remain deeply embedded, despite broader changes in health systems and increased attention to nursing. This persistence suggests that while nursing has evolved in scope and professional recognition, societal attitudes towards gender roles within the profession have not progressed at the same pace.

These findings suggest that recruitment strategies could seek to challenge these stereotypes by focusing on redefining nursing as a scientific, inclusive, and gender‐neutral profession. Similar to how stay‐at‐home fathers redefine masculine identities through caregiving roles, nursing can also be repositioned as a profession that integrates qualities of care and leadership, promoting inclusivity across genders (Lee and Lee 2018). Media campaigns could portray gender equality and diversity in nursing, such as by showcasing male nurses in leadership roles to challenge traditional perceptions of nursing image. Educational institutions could consider highlighting nursing's critical thinking and decision‐making components and develop outreach programs to improve pre‐nursing students’ perceptions of nursing as a profession.

### 5.2 Managing Parents’ Expectations and Countering Peer‐Driven Influence

Managing parents’ expectations and countering peer‐driven influence remain central in shaping pre‐nursing students’ perceptions of nursing image. According to Smetana and Rote (2019), autonomy negotiation between parents and youths often shapes how parental views are internalized during adolescence. Supportive and autonomy‐encouraging parenting allows open discussion and independent exploration, while controlling or status‐oriented parenting reinforces stereotypes and deters interest in nursing. Dialogues that involve both parents and students can help reframe nursing as a profession that is prestigious and rewarding, thereby countering negative perceptions.

Similarly, peer influence exerts equally powerful pressure. During their formative years, adolescents are especially responsive to peer validation as the need for belonging intensifies during this stage (Ciranka and Van den Bos 2019; Defoe et al. 2018; Steinberg and Monahan 2007). Negative peer feedback about nursing can therefore discourage students from viewing it as desirable, as adolescents are likely to conform to group expectations. This pattern is consistent with social identity theory, which posits that peer approval plays a central role in identity formation during this period (Tajfel 1978).

Evidence across studies and cultural contexts suggests that such influence operates not only through direct interactions but also through shared cultural understandings of appropriate behavior (Murray 2002; Zamanzadeh et al. 2023). However, the relative weight of these influences may vary by context: collectivist settings often place greater emphasis on family approval, social reputation, and gender norms, whereas individualistic contexts tend to prioritize personal fit, autonomy, and perceived career benefits (Triandis 2018).

Given this, addressing peer influence requires interventions embedded within youth social networks rather than focusing solely on individual attitudes. Peer‐led workshops, which are defined as young people educating young people (Shiner 1999), have been widely used with adolescents in health education (Dodd et al. 2022) and have demonstrated significant effectiveness in addressing issues heavily influenced by stereotypes, such as sexual health, substance abuse and mental health (MacArthur et al. 2016; Maticka‐Tyndale and Barnett 2010; Siddiqui et al. 2020). Extending this approach to recruitment strategies may help challenge stereotypes and foster more positive perceptions of nursing image. Importantly, such interventions should remain context‐sensitive, considering local cultural and gender norms that shape how nursing is perceived.

### 5.3 Addressing Knowledge Gaps Among Students Through Exposure

Despite nurses’ heightened visibility during the COVID‐19 pandemic, persistent stereotypes indicate that passive exposure through media and public discourse alone is insufficient to shift entrenched views. Studies have consistently identified educational gaps as contributors to an overly simplistic view of nursing roles (Cabaniss 2011; Godsey et al. 2020; Price and McGillis Hall 2014).

Only 6 out of 32 studies in this review were interventional trials aimed explicitly at improving the nursing image. Drawing on experiential learning theory (Kolb 1984), structured programs such as hospital visits and job‐shadowing may help address students’ knowledge gaps by providing firsthand exposure to nursing roles. Such experiential exposure helps make abstract concepts about the profession more concrete and accessible (Linder and Pulsipher 2008). By observing nurses’ roles, professional interactions, and decision‐making processes, students gain a clearer understanding of the complexity and scope of nursing. These experiences should highlight nursing's intellectual and leadership responsibilities, rather than focusing solely on caregiving tasks to challenge existing negative perceptions.

### 5.4 Enhancing Nursing Visibility Through Leadership and Media Portrayals

The limited visibility of nursing's leadership and intellectual roles shapes societal understanding of nursing, often reducing recognition of nurses’ expertise and authority. In this review, findings showed that media portrayals contribute substantially to this phenomenon. Although prior research has emphasized the media's influence in shaping public perceptions of health professions (Huber et al. 2020; Lian and Dong 2021), the pre‐2020 studies included in this review suggest that interactive media tools and campaigns can promote a more dynamic understanding of health professions, although this evidence predates the pandemic, and their effectiveness for contemporary recruitment has not been established (Drenkard et al. 2002; Droes et al. 1993; Matutina 2012; Tawash and Cowman 2018).

Over the years, media approaches have evolved from traditional 2D and participative media interventions to interactive and immersive 3D virtual reality (Scolari 2023). Given the growing reliance on digital media among Generation Z, recruitment strategies could consider embracing innovative, technology‐driven approaches that resonate with the expectations of today's society (Tomčíková et al. 2021). The pandemic has shown that traditional recruitment methods, such as in‐person events and legacy tactics like purchasing contact lists, are no longer sufficient (Saini and Tarkar 2023). Instead, institutions could consider piloting immersive tools to provide prospective students with realistic insights into nursing roles, showcasing the profession's intellectual and leadership dimensions.

At the same time, findings from post‐COVID studies in this review highlight the double‐edged nature of “hero” narratives. While students and young adults admired nurses’ courage and lifesaving work, they also linked nursing with fatigue, difficult working conditions, and limited independence. This supports wider critiques that hero framing can normalize self‐sacrifice while leaving understaffing, low pay, and unsafe environments unaddressed (Mohammed et al. 2021; Stanley and Kay 2024). Media and digital strategies may therefore benefit from combining compelling stories with depictions of working realities and advocacy for structural improvements, so that nursing is presented as a knowledge‐intensive and well‐supported profession.

## 6 Implications for Nursing and Health Policy

This review highlights critical gaps in research and practice regarding pre‐nursing students’ perceptions of the nursing image. Given the impact of the COVID‐19 pandemic and evolving societal expectations, policymakers, educators, healthcare administrators, and researchers may consider coordinated approaches in shaping a more accurate nursing image and strengthening nursing recruitment.

At the national level, policymakers could consider supporting national campaigns that challenge gender stereotypes and promote nursing as a dynamic and intellectually rigorous profession. Next, at the educational level, nursing educators should integrate experiential learning opportunities into career education for pre‐nursing students. Career talks could also incorporate parental engagement programs to address misconceptions and emphasize career progression, intellectual rigor, and leadership roles. Additionally, peer‐led workshops have been effective in addressing stigma in other fields such as mental health and substance abuse education. Implementing similar approaches in nursing recruitment could challenge prevailing stereotypes and encourage positive peer discussions around nursing as a profession.

Furthermore, at the healthcare institutional level, hospitals and healthcare organizations could contribute by partnering with schools for career talks, internship programs, and media campaigns featuring real nurses in leadership and specialized roles. Given the limited interventional evidence, these recommendations should be implemented in phases according to scalability and resource requirements. Low‐resource strategies that can be embedded within existing education and outreach structures should be prioritized first. More intensive experiential or technology‐enhanced strategies may offer richer exposure but require greater institutional support and should be piloted and evaluated before wider implementation. Potential barriers to implementation include competing institutional priorities, limited availability of nursing ambassadors, scheduling constraints within school curricula, and variability in stakeholder engagement across different school and healthcare settings. These should be anticipated and addressed in the planning phase of any recruitment initiative.

Lastly, at the research level, this review identified that only 7 out of 32 primary studies (*k* = 7, 21.9%) were conducted in the past five years, indicating a significant gap in recent research exploring pre‐nursing students’ perceptions of the nursing image. Given the limited recent evidence, further exploratory studies are needed to examine post‐pandemic and context‐specific factors influencing nursing image among this population. Future research should also explore innovative interventions in shaping students’ perceptions. Evaluating these interventions will provide evidence‐based recommendations to refine strategies aimed at improving nursing image and ensure that efforts remain relevant.

## 7 Strengths and Limitations

To our knowledge, this is the first mixed‐methods systematic review that examined factors influencing pre‐nursing students’ perceptions of nursing image. By synthesizing findings from quantitative, qualitative, and mixed‐methods studies, it provides a more nuanced and comprehensive understanding of this phenomenon. The use of Bronfenbrenner's SEM further strengthened the analysis across individual, interpersonal, and societal levels, enabling a detailed exploration of overlapping influences. The inclusion of studies from diverse regions, including high‐income countries and parts of Asia and the Middle East, allows for meaningful cross‐cultural insights into the global context of nursing image.

Despite its strengths, a major limitation of this review is the language restriction to English and Mandarin sources. Although this broadened eligibility beyond English‐language literature, relevant studies published in other languages may have been missed, particularly from regions where nursing image may be shaped by different sociocultural and professional contexts. This introduces potential language bias and may limit the comprehensiveness and transferability of the findings.

A further limitation is the temporal distribution of the included studies. Only 7 of the 32 studies were published from 2020 onward, while the remaining evidence predates shifts in healthcare visibility associated with the COVID‐19 pandemic. The post‐2020 studies confirm that several long‐standing perceptions persist, including limited understanding of nursing roles, gendered perceptions of nursing, sociocultural barriers, perceived low status, demanding working conditions, and limited perceived autonomy or career advancement. They also add recent evidence that increased pandemic‐related visibility may improve recognition of nurses without necessarily resolving entrenched stereotypes. In contrast, evidence on targeted recruitment interventions, including camps, orientation days, career talks, media‐based campaigns, and ambassador‐led programs, was drawn entirely from studies published before 2020. Therefore, claims about contemporary perceptions and the effectiveness of recruitment strategies should be interpreted cautiously until more recent, theory‐driven, and interventional studies are available.

Nearly half of the included studies were conducted in the United States, which may have overrepresented Western perspectives. This limits the extent to which the findings can capture how nursing image is shaped in non‐Western, post‐colonial, or low‐resource contexts, where social norms and the public standing of nursing may differ substantially. High heterogeneity among quantitative findings precluded a meta‐analysis, so findings were synthesized narratively to identify consistent patterns rather than pooled estimates. Several synthesized factors showed no significant association, which may reflect uncertainty rather than true neutrality and therefore warrant further investigation in future studies. Few studies were interventional or theory‐driven, and many relied on researcher‐developed instruments without established validity, reducing comparability across studies. Only three qualitative studies were identified, limiting our understanding of cultural and personal influences. Furthermore, the cross‐sectional design of the included studies limits conclusions about whether improved perceptions lead to sustained outcomes such as nursing program enrolment. Reporting of participants’ SES was also inconsistent. Most studies did not report SES, race/ethnicity, or school type in a consistent format, which limited analysis of how overlapping social positions shaped perceptions of nursing image. Evidence indicates that SES affects perceived work attributes of nursing (Li et al. 2023), and the absence of SES data limits interpretation of potential differences in perceptions of nursing image. These limitations highlight the need for future research to expand the scope of inquiry, include more studies from non‐Western and low‐resource settings, incorporate non‐English literature where possible, report SES more consistently, and conduct more interventional studies to build a stronger evidence base.

## 8 Conclusion

In conclusion, this review highlights the importance of understanding the factors influencing pre‐nursing students’ perceptions of nursing image at three levels: individual, interpersonal, and societal. Targeted interventions such as educational programs, peer and family engagement initiatives, and strategic media campaigns showed potential to reshape perceptions in the studies reviewed, though this evidence predates 2020 and remains limited. Future studies should incorporate longitudinal designs, include diverse cultural and economic contexts, and prioritize interventional approaches to strengthen the evidence base. A focused and sustained effort to improve pre‐nursing students’ perceptions of nursing image, in turn, supports broader efforts to address the global nursing shortage by creating a more informed and receptive pool of prospective students.

## Author Contributions

Study design: LSS, JG, and LSY. Data collection: LSS and JG. Data analysis: LSS and JG. Study supervision: LSY. Manuscript writing: LSS. Critical revisions for important intellectual content: LSS, JG, SL, IR, VL, and LSY.

## Conflicts of Interest

The authors declare no conflicts of interest.

## Ethics Statement

This study did not involve human participants or animals and is exempt from ethical approval.

## Funding

The authors have nothing to report.

## Supporting information




**Table S1**: Full Search Strategy for All Databases.
**Table S2**: Eligibility criteria for study selection.
**Table S3**: Illustrative examples of integration of quantitative and qualitative evidence across Social–Ecological Model levels.
**Table S4**: List of excluded articles with reasons.
**Table S5**: Data Extraction Table.
**Table S6**: Geographic distribution of included studies.
**Table S7**: Summary of quantitative findings.
**Table S8**: Quality Assessment Results Mixed‐Methods Appraisal Tool.
