# Supplementary material for: Nursing Image Among Pre‐Nursing Students: A Mixed‐Methods Systematic Review with Future Directions on Recruitment Strategies
Source: Int Nurs Rev. 2026 Sep 25;73(3):e70234. doi: 10.1111/inr.70234 (PMC13613994; doi:10.1111/inr.70234)
Supplement: Supplementary file 1 [file INR-73-0-s001.docx]

**Table S1** *Full Search Strategy for All Databases*

| **Database** | **Index and keyword terms used in the database** |
| --- | --- |
| Pubmed | (("Adolescent"[Mesh] OR "Young Adult"[Mesh] OR "Students, Nursing"[Mesh] OR "Adolescen*"[tiab] OR Youth*[Title/Abstract] OR Teen*[Title/Abstract] OR Teenager*[Title/Abstract] OR Young Adult*[Title/Abstract] OR "middle school"[Title/Abstract] OR "high school"[Title/Abstract] OR "Nursing student*"[tiab]) AND  ("Health Knowledge, Attitudes, Practice"[Mesh] OR "Social Values"[Mesh] OR "Career Choice"[Mesh] OR "Social Perception"[Mesh] OR "Marketing of Health Services"[Mesh] OR perception*[tiab] OR attitude*[tiab] OR portray*[tiab] OR depict*[tiab] OR perceive*[tiab] OR recognition*[tiab] OR recogni*[tiab] OR career choice*[tiab] OR "career choice"[tiab:~3] OR Motivation*[tiab] OR Disincentive*[tiab] OR Incentive*[tiab] OR Expectation*[tiab]))  AND  ((("Nurses"[Mesh] OR "Nurse's Role"[Mesh] OR "Nursing"[Mesh] OR "Legislation, Nursing"[Mesh] OR "Nursing Staff"[Mesh] OR Nurse[tiab] OR nurses[tiab] OR nursing[tiab] OR Nursing staff*[tiab]) AND (Image* OR social image[tiab] OR social status[tiab] OR status[tiab] OR stereotype*[tiab] OR career[tiab])) OR (Nursing image[tiab] OR "nursing image"[tiab:~3] OR nurse image[tiab] OR "nurse image"[tiab:~3] OR nursing career[tiab] OR "nursing career"[tiab:~3])) |
| Medline (Ovid) | exp Adolescent/ or exp Young Adult/ or exp Students, Nursing/ or (Adolescen* OR Youth* OR Teen* OR Teenager* OR Young Adult* OR middle school OR high school OR Nursing student*).mp AND exp Health Knowledge, Attitudes, Practice/ OR exp Social Values/ OR exp Career Choice/ OR exp Social Perception/ OR exp Marketing of Health Services/ OR (perception* OR attitude* OR portray* OR depict* OR perceive* OR recognition* OR recogni* OR career choice* OR career ADJ3 choice* OR Motivation* OR Disincentive* OR Incentive* OR Expectation*).mp AND  ((exp Nurses/ OR exp Nurse’s Role/ OR exp Nursing/ OR exp Legislation, Nursing/ OR exp Nursing Staff/ OR (Nurse* OR nursing OR nursing staff).mp) AND (Image* OR social image OR social status OR status OR stereotype* OR career).mp) OR (Nursing Image OR Nursing ADJ3 Image OR Nurse Image OR Nurse ADJ3 Image OR Nursing career OR Nursing ADJ3 Career) |
| Embase | ('college student'/exp OR 'high school student'/exp OR 'middle school student'/exp OR 'non-medical student'/exp OR 'undergraduate student'/exp OR 'university student'/exp OR 'nursing student'/exp OR 'adolescent'/exp OR 'young adult'/exp OR ('apprentice' OR 'honor student' OR 'honour student' OR 'student' OR 'students' OR 'trainee' OR 'health care student' OR 'health occupation* student*' OR 'health student' OR 'healthcare student' OR 'students, health occupations' OR student OR 'juvenile' OR 'youth' OR 'adolescent' OR 'teenager' OR 'middle school student'):ti,ab)  AND  ('attitude to health'/exp OR 'social value'/exp OR 'decision making'/exp OR 'perception'/exp OR ('attitude to health' OR 'attitudes to health' OR 'health attitude' OR 'health attitudes and knowledge' OR 'health knowledge' OR 'health knowledge and attitude' OR 'health knowledge and attitudes' OR 'health knowledge, attitudes, practice' OR 'arbitration' OR 'career choice' OR 'decision making' OR 'decision process' OR 'decisionmaking' OR 'judgement' OR 'judgment' OR 'perception*' OR 'social perception' OR 'marketing of health services'):ti,ab)  AND  (('nursing'/exp OR 'stereotypy'/exp OR 'career'/exp) OR ('hospital nursing service' OR 'nursing' OR 'nursing service' OR 'nursing service, hospital' OR 'nursing services' OR 'nursing support' OR 'nursing, private duty' OR 'nursing, supervisory' OR 'office nursing' OR 'private duty nursing' OR 'supervisory nursing'):ti,ab) AND (('social status'/exp) OR ('social achievement' OR 'social condition*’ OR 'social importance' OR 'social rank' OR 'social standing' OR 'social state' OR 'social status' OR 'status, social'):ti,ab) |
| Cinahl | ((MM "Students, College+") OR (MM "Students, High School") OR (MM "Students, Midwifery") OR (MM "Students, Nursing+") OR (MM "Students, Nursing, Male") OR (MM "Students, Nursing, Graduate+") OR (MM "Students, Undergraduate") OR (MH "Students, Nursing, Associate") OR (MM "Students, Nurse Midwifery") OR (MM "Students, Pre-Nursing") OR (MM "Students, Nursing, Masters") OR (MM "Students, Nursing, Baccalaureate") OR (MM "Students, Nursing, Diploma Programs") OR (MM "Students+") OR (MM "Students, Middle School") OR (MM "Students, Health Occupations+") OR (MM "Young Adult") OR (MM "Adolescence+") OR "student") OR TI (Adolescen* OR Youth* OR Teen* OR Teenager* OR Young Adult* OR middle school OR high school OR Nursing student*) OR AB (Adolescen* OR Youth* OR Teen* OR Teenager* OR Young Adult* OR middle school OR high school OR Nursing student*))  AND  ((MM "Student Recruitment") OR (MM "Student Attitudes+") OR (MM "Student Knowledge") OR (MM "Health Knowledge") OR (MM "Student Knowledge") OR (MM "Nursing Knowledge") OR (MM "Knowledge+") OR (MM "Perception+") OR (MM "Social Perception+") OR (MM "Social Values+") OR (MM "Careers in Nursing") OR TI (perception* OR attitude* OR portray* OR depict* OR perceive* OR recognition* OR recogni* OR career choice* OR Motivation* OR Disincentive* OR Incentive* OR Expectation*) OR AB (perception* OR attitude* OR portray* OR depict* OR perceive* OR recognition* OR recogni* OR career choice* OR Motivation* OR Disincentive* OR Incentive* OR Expectation*)) AND  (((MM "Nurses+") OR (MM "Faculty, Nursing") OR (MM "Frontline Nurses") OR (MM "Nurses byEducational Level+") OR (MM "Nurses by Role+") OR (MM "Nursing Home Personnel") OR (MM "Operating Room Personnel+") OR TI (Nurse* OR nursing OR Nursing staff*) OR AB (Nurse* OR nursing OR Nursing staff*)) AND ((MM "Professional Image") OR TI (Image* OR social image OR social status OR status OR stereotype* OR career) OR AB (Image* OR social image OR social status OR status OR stereotype* OR career)) OR TI (Nursing image OR nursing image OR nursing career) OR AB (Nursing image OR nursing image OR nursing career) OR TI ((Nurse* OR Nursing) W3 (Image OR career)) OR AB ((Nurse* OR Nursing) W3 (Image OR career)) |
| Web of science | TI = (Adolescen* OR Youth* OR Teen* OR Teenager* OR Young Adult* OR middle school OR high school OR Nursing student*) OR AB=(Adolescen* OR Youth* OR Teen* OR Teenager* OR Young Adult* OR middle school OR high school OR Nursing student*)  AND  TI = (perception* OR attitude* OR portray* OR depict* OR perceive* OR recognition* OR recogni* OR career choice* OR Motivation* OR Disincentive* OR Incentive* OR Expectation*) OR AB=(perception* OR attitude* OR portray* OR depict* OR perceive* OR recognition* OR recogni* OR career choice* OR Motivation* OR Disincentive* OR Incentive* OR Expectation*)  AND  (TI = (Nurse* OR nursing OR Nursing staff*) OR AB = (Nurse* OR nursing OR Nursing staff*)) AND ((TI = (Image* OR social image OR social status OR status OR stereotype* OR career) OR AB = (Image* OR social image OR social status OR status OR stereotype* OR career) OR TI = (Nursing image OR nursing image OR nursing career) OR AB = (Nursing image OR nursing image OR nursing career))) |
| ERIC | subject("Adolescents" OR “Young adults” OR “youth” OR “teen” OR “middle school” OR “high school” OR “student” OR “nursing student") OR title(Adolescen* OR Youth* OR Teen* OR Teenager* OR Young Adult* OR middle school OR high school OR Nursing student*) OR abstract(Adolescen* OR Youth* OR Teen* OR Teenager* OR Young Adult* OR middle school OR high school OR Nursing student*)  AND  subject("recruitment" OR "attitude*" OR "knowledge" OR "perception*" OR "social value*" OR "career choice" OR "nursing career") OR title (perception* OR attitude* OR portray* OR depict* OR perceive* OR recognition* OR recogni* OR career choice* OR Motivation* OR Disincentive* OR Incentive* OR Expectation*) OR Abstract (perception* OR attitude* OR portray* OR depict* OR perceive* OR recognition* OR recogni* OR career choice* OR Motivation* OR Disincentive* OR Incentive* OR Expectation*)  AND  (((subject("nurse*" OR "nursing") OR Title (Nurse* OR nursing OR Nursing staff*) OR abstract (Nurse* OR nursing OR Nursing staff*)) AND (title (Image* OR social image OR social status OR status OR stereotype* OR career) OR Abstract (Image* OR social image OR social status OR status OR stereotype* OR career))) OR Title (Nursing image OR nursing image OR nursing career) OR abstract (Nursing image OR nursing career) OR Title ((Nurse* OR Nursing) N3 (Image OR career)) OR Abstract ((Nurse* OR Nursing) N3 (Image OR career))) |
| Scopus | TITLE-ABS-KEY (Adolescen* OR Youth* OR Teen* OR Teenager* OR Young Adult* OR middle school OR high school OR Nursing student*)  AND  TITLE-ABS-KEY (perception* OR attitude* OR portray* OR depict* OR perceive* OR recognition* OR recogni* OR career choice* OR Motivation* OR Disincentive* OR Incentive* OR Expectation*)  AND  TITLE-ABS-KEY ((Nurse* OR nursing OR Nursing staff*) AND (Image* OR social image OR social status OR status OR stereotype* OR career)) OR Nursing image OR nursing career OR ((nurse* OR nursing ) W/3 ( image OR career)) |
| PsycINFO | exp Adolescen$/ or exp Young Adult/ or exp Students, Nursing/ or (Adolescen$ OR Youth$ OR Teen$ OR Teenager$ OR Young Adult$ OR middle school OR high school OR Nursing student$).ab,ti,tw. AND exp Social Values/ or exp Career Choice/ or exp Social Perception/ or exp Marketing of Health Services/ or (((perception$ or attitude$ or portray$ or depict$ or perceive$ or recognition$ or recogni$ or career choice$ or career) adj3 choice$) or Motivation$ or Disincentive$ or Incentive$ or Expectation$).ab,ti,tw.  AND  ((exp Nurses/ or exp Nursing/ or exp Legislation, Nursing/ or exp Nursing Staff/ OR (Nurse$ OR nursing OR nursing staff).ab,ti,tw.) AND ((Image$ OR social image OR social status OR status OR stereotype$ OR career).ab,ti,tw.) OR Nursing Image OR Nurse Image OR Nursing ADJ3 career OR Nurse ADJ3 Career) |
| ProQuest Dissertations & Theses Global | Mainsubject(Adolescent) OR Mainsubject(Young Adult) OR Mainsubject(Students, Nursing) OR ti(Adolescen$ OR Youth$ OR Teen$ OR Teenager$ OR Young Adult$ OR middle school OR high school OR “Nursing student$”) OR ab(Adolescen$ OR Youth$ OR Teen$ OR Teenager$ OR Young Adult$ OR middle school OR high school OR “Nursing student$”)  AND  Mainsubject(Health Knowledge, Attitudes, Practice) OR Mainsubject(Social Values) OR Mainsubject(Career Choice) OR Mainsubject(Social Perception) OR Mainsubject(Marketing of Health Services) OR ti(perception$ OR attitude$ OR portray$ OR depict$ OR perceive$ OR recognition$ OR recogni$ OR career choice$ OR Motivation$ OR Disincentive$ OR Incentive$ OR Expectation$) Or ab(perception$ OR attitude$ OR portray$ OR depict$ OR perceive$ OR recognition$ OR recogni$ OR career choice$ OR Motivation$ OR Disincentive$ OR Incentive$ OR Expectation$)  AND  ((Mainsubject(Nursing) OR Mainsubject(Legislation, Nursing) OR Mainsubject(Nursing Staff) OR ti(Nurse$ OR nursing OR Nursing staff$) OR ab(Nurse$ OR nursing OR Nursing staff$)) AND (ti(Image$ OR social image OR social status OR status OR stereotype$ OR career) OR ab(Image$ OR social image OR social status OR status OR stereotype$ OR career)) OR ti(Nursing image OR nursing career) OR ab(Nursing image OR nursing career) OR ti(nurs$ image OR Nurs$ career) OR ab(nurs$ image OR nurs$ career)) |

**Table S2** *Eligibility criteria for study selection.*

| **Eligibility Criteria** | **Inclusion** | **Exclusion** |
| --- | --- | --- |
| Population | - Pre-nursing students who had not commenced nursing education or entered nursing practice | - Practicing nurse - Nursing students (including diploma and undergraduate) - Other healthcare students or professionals |
| Exposure | - Exploratory studies identifying factors influencing perceptions of the nursing image |  |
| Outcome | - Individual level factors - Interpersonal Relationship level factors - Community/Societal level factors |  |
| Outcome (Secondary) | - Cross domains interactions |  |
| Types of sources | - Original articles reporting the evaluation of the perceptions towards nursing image - Quantitative - Qualitative - Mixed-Methods | - Systematic review |
| Publication type | - Published trials - Unpublished trials | - Book chapter review - Letters - Editorials - Magazine - Commentaries |
| Language | - English or Mandarin | - Non-English or Non-Mandarin |
| Years of publication | - No limit |  |

**Table S3** *Illustrative examples of integration of quantitative and qualitative evidence across Social Ecological Model levels*

| **SEM Level** | **Factor** | **Quantitative evidence** | **Qualitative evidence** | **Integration outcome** |
| --- | --- | --- | --- | --- |
| Individual | Gender Stereotypes | Andrews (2005) found that high-school boys generally perceived nursing positively in terms of prestige but gender-typed it as a woman’s occupation. | Hemsley-Brown & Foskett (1999) linked this to gender-role stigma, illustrated by a male participant who avoided nursing to prevent being perceived as gay. Zamanzadeh et al. (2023) found female students associated nursing with caregiving while male students viewed it as intellectually limiting. | Expanded. Qualitative data explained the mechanism behind the pattern. |
| Interpersonal | Exposure to Nursing Professionals | Rossiter et al. (1999) found more favourable attitude scores among students acquainted with a nurse, p = .013, although knowledge scores did not differ, p = .294. | Hospital exposure reinforced a positive view in one qualitative account (Zamanzadeh et al., 2023). Murray (2002) described both positive and negative accounts of students’ personal experiences with nurses. | Expanded. The qualitative account illustrated how observing nurses’ work could contribute to the more favourable attitudes associated with professional exposure, while the quantitative evidence showed that exposure did not necessarily improve knowledge. |
| Societal | Targeted Recruitment Interventions | Droes et al. (1993) reported significant intervention effects for caring, F = 20.17, usefulness, F = 13.18, autonomy, F = 38.85, scientific knowledge, F = 34.75, and total nursing-image score, F = 42.72, all p = 0.001 | Analysis of open-ended responses in Raymond et al. (2018) showed that students initially understood nursing mainly through caring and clinical tasks. Following the promotional programme, students identified a wider range of nursing and midwifery roles, and 47 participants reported learning about roles of which they had previously been unaware. | Confirmed and expanded. The quantitative evidence showed that targeted exposure could improve specific nursing-image dimensions, while the mixed-methods evidence suggested that broadening awareness of the diversity of nursing roles was one mechanism through which such interventions could reshape perceptions. |

Note. Confirmed = qualitative and quantitative evidence describe the same pattern. Expanded = qualitative evidence adds a mechanism, nuance, or boundary condition not captured quantitatively. No factor met the criterion for Contradicted.

**Table S4** *List of excluded articles with reasons.*

| Year of Publication | Identification | Reference of excluded studies | Reason for exclusion |
| --- | --- | --- | --- |
| 2008 | Databases and registers | Albert, N. M., Wocial, L., Meyer, K. H., Na, J., & Trochelman, K. (2008). Impact of nurses' uniforms on patient and family perceptions of nurse professionalism. *Appl Nurs Res*, *21*(4), 181-190. https://doi.org/10.1016/j.apnr.2007.04.008 | Wrong population |
| 2015 | Databases and registers | Hadid, S., & Khatib, M. (2015). The public's perception of the status and image of the nursing profession. *Med Law*, *34*(1), 69-90. | Wrong population |
| 1999 | Databases and registers | Happell, B. (1999). The image of critical care nursing: does it tell the whole story? *Aust Crit Care*, *12*(2), 55-58. https://doi.org/10.1016/s1036-7314(99)70537-0 | Wrong population |
| 1994 | Databases and registers | Pillitteri, A. (1994). A contrast in images: nursing and nonnursing college students. *J Nurs Educ*, *33*(3), 132-133. https://doi.org/10.3928/0148-4834-19940301-09 | Wrong population |
| 2006 | Databases and registers | Seago, J. A., Spetz, J., Alvarado, A., Keane, D., & Grumbach, K. (2006). The nursing shortage: is it really about image? *J Healthc Manag*, *51*(2), 96-108; discussion 109-110. | Wrong population |
| 1989 | Databases and registers | Kaler, S. R., Levy, D. A., & Schall, M. (1989). Stereotypes of professional roles. *Image J Nurs Sch*, *21*(2), 85-89. https://doi.org/10.1111/j.1547-5069.1989.tb00104.x | Wrong population |
| 2024 | Databases and registers | Dahlke, S., Rayner, J. A., Fetherstonhaugh, D., Butler, J. I., & Kennedy, M. (2025). Gerontological educational interventions for student nurses: a systematic review of qualitative findings. *Int J Nurs Educ Scholarsh*, *22*(1). https://doi.org/10.1515/ijnes-2023-0042 | Wrong population |
| 2023 | Databases and registers | Dolu, İ., Gökçe, M. C., Karakuş, E., Alperen Geldi, O., & Gazi, T. (2023). How do male nursing students' voluntary activities affect their image in society? *Int Nurs Rev*, *70*(3), 363-371. https://doi.org/10.1111/inr.12821 | Wrong population |
| 1986 | Databases and registers | Bzdek, V., Ganong, L. H., & Douglas, D. C. (1986). Images of nursing in Missouri: a test of Kalisch's and Kalisch's typology. *Mo Nurse*, *55*(5), 10-11. | Wrong population |
| 1997 | Databases and registers | Mangum, S., Garrison, C., Lind, C., & Hilton, H. G. (1997). First impressions of the nurse and nursing care. *J Nurs Care Qual*, *11*(5), 39-47. https://doi.org/10.1097/00001786-199706000-00009 | Wrong population |
| 2005 | Databases and registers | Rudland, J. R., & Mires, G. J. (2005). Characteristics of doctors and nurses as perceived by students entering medical school: implications for shared teaching. *Medical Education*, *39*(5), 448-455. https://doi.org/10.1111/j.1365-2929.2005.02108.x | Wrong population |
| 2009 | Databases and registers | Marcinowicz, L., Foley, M., Zarzycka, D., Chlabicz, S., Windak, A., & Buczkowski, K. (2009). Polish medical students' perceptions of the nursing profession: a cross-sectional study. *Scand J Caring Sci*, *23*(3), 438-445. https://doi.org/10.1111/j.1471-6712.2008.00638.x | Wrong population |
| 2018 | Databases and registers | Alkaya, S. A., Yaman, Ş., & Simones, J. (2018). Professional values and career choice of nursing students. *Nurs Ethics*, *25*(2), 243-252. https://doi.org/10.1177/0969733017707007 | Wrong population |
| 2018 | Databases and registers | Riklikiene, O., Karosas, L., & Kaseliene, S. (2018). General and professional values of student nurses and nurse educators. *J Adv Nurs*, *74*(3), 666-676. https://doi.org/10.1111/jan.13473 | Wrong population |
| 2019 | Databases and registers | Marcinowicz, L., Andersson, E. K., Bohman, D. M., Hjelm, M., Skarbalienė, A., Shpakou, A., Kalinowska, P., & Jamiolkowski, J. (2019). Nursing students' perception of the professional nurse's role in four European countries. *Int Nurs Rev*, *66*(2), 250-258. https://doi.org/10.1111/inr.12494 | Wrong population |
| 2019 | Databases and registers | Çöplü, M., & Tekinsoy Kartın, P. (2019). Professional self-concept and professional values of senior students of the nursing department. *Nurs Ethics*, *26*(5), 1387-1397. https://doi.org/10.1177/0969733018761171 | Wrong population |
| 2016 | Databases and registers | Ten Hoeve, Y., Castelein, S., Jansen, W., Jansen, G., & Roodbol, P. (2016). Predicting factors of positive orientation and attitudes towards nursing: A quantitative cross-sectional study. *Nurse Educ Today*, *40*, 111-117. https://doi.org/10.1016/j.nedt.2016.02.019 | Wrong population |
| 2009 | Databases and registers | Ben Natan, M. (2009). Effects of nursing education on the image of nursing as a profession in Israel. *Int J Nurs Educ Scholarsh*, *6*, Article 38. https://doi.org/10.2202/1548-923x.1942 | Wrong population |
| 2015 | Databases and registers | Cowin, L. S., & Johnson, M. (2015). From student to graduate: longitudinal changes in the qualities of nurses. *J Adv Nurs*, *71*(12), 2911-2922. https://doi.org/10.1111/jan.12763 | Wrong population |
| 2017 | Databases and registers | Cukljek, S., Juresa, V., & Babic, J. (2017). The cross-cultural (transcultural) adaptation and validation of the nursing image questionnaire. *Nurse Educ Today*, *48*, 67-71. https://doi.org/10.1016/j.nedt.2016.09.006 | Wrong population |
| 2012 | Databases and registers | Poreddi, V., Ramachandra, Konduru, R., & Math, S. B. (2012). Assessing the attitudes and perceptions towards nursing profession among nursing students. *Nurs J India*, *103*(1), 6-8. | Wrong population |
| 2020 | Databases and registers | Nocerino, R., Chiarini, M., & Marina, M. (2020). Nurse professional identity: Validation of the Italian version of the questionnaire Nurse Professional Values Scale-Revised. *Clin Ter*, *171*(2), e114-e119. https://doi.org/10.7417/ct.2020.2200 | Wrong population |
| 2015 | Databases and registers | Fillman, V. M. (2015). Career interest, self-efficacy, and perception in undecided and nursing undergraduate students: a quantitative study. *Nurse Educ Today*, *35*(1), 251-255. https://doi.org/10.1016/j.nedt.2014.08.011 | Wrong population |
| 2004 | Databases and registers | Brodie, D. A., Andrews, G. J., Andrews, J. P., Thomas, G. B., Wong, J., & Rixon, L. (2004). Perceptions of nursing: confirmation, change and the student experience. *Int J Nurs Stud*, *41*(7), 721-733. https://doi.org/10.1016/j.ijnurstu.2004.02.009 | Wrong population |
| 2006 | Databases and registers | Tzeng, H. M. (2006). Testing a conceptual model of the image of nursing in Taiwan. *Int J Nurs Stud*, *43*(6), 755-765. https://doi.org/10.1016/j.ijnurstu.2005.10.004 | Wrong population |
| 2013 | Databases and registers | Weaver, R., Salamonson, Y., Koch, J., & Jackson, D. (2013). Nursing on television: student perceptions of television's role in public image, recruitment and education. *J Adv Nurs*, *69*(12), 2635-2643. https://doi.org/10.1111/jan.12148 | Wrong population |
| 2019 | Databases and registers | van den Boogaard, T. C., Roodbol, P. F., Poslawsky, I. E., & Ten Hoeve, Y. (2019). The orientation and attitudes of intermediate vocational trained nursing students (MBO-V) towards their future profession: A pre-post survey. *Nurse Educ Pract*, *37*, 124-131. https://doi.org/10.1016/j.nepr.2019.04.007 | Wrong population |
| 2006 | Databases and registers | Grainger, P., & Bolan, C. (2006). Perceptions of nursing as a career choice of students in the Baccalaureate nursing program. *Nurse Educ Today*, *26*(1), 38-44. https://doi.org/10.1016/j.nedt.2005.06.004 | Wrong population |
| 2011 | Databases and registers | Hendel, T., & Kagan, I. (2011). Professional image and intention to emigrate among Israeli nurses and nursing students. *Nurse Educ Today*, *31*(3), 259-262. https://doi.org/10.1016/j.nedt.2010.11.008 | Wrong population |
| 2010 | Databases and registers | Milisen, K., De Busser, T., Kayaert, A., Abraham, I., & de Casterlé, B. D. (2010). The evolving professional nursing self-image of students in baccalaureate programs: a cross-sectional survey. *Int J Nurs Stud*, *47*(6), 688-698. https://doi.org/10.1016/j.ijnurstu.2009.11.008 | Wrong population |
| 2012 | Databases and registers | Patidar, A. B., Kaur, J., & Sharma, S. (2012). Perception and plans about profession among budding nurses: a descriptive survey. *Nurs J India*, *103*(5), 221-225. | Wrong population |
| 1991 | Databases and registers | Mendez, D., & Louis, M. (1991). College students' image of nursing as a career choice. *J Nurs Educ*, *30*(7), 311-319. https://doi.org/10.3928/0148-4834-19910901-07 | Wrong population |
| 2003 | Databases and registers | Ward, C., Styles, I., & Bosco, A. M. (2003). Perceived status of nurses compared to other health care professionals. *Contemp Nurse*, *15*(1-2), 20-28. https://doi.org/10.5172/conu.15.1-2.20 | Wrong population |
| 2019 | Databases and registers | Hung, C. A., Wu, P. L., Liu, N. Y., Hsu, W. Y., Lee, B. O., & Pai, H. C. (2019). The effect of gender-friendliness barriers on perceived image in nursing and caring behaviour among male nursing students. *J Clin Nurs*, *28*(9-10), 1465-1472. https://doi.org/10.1111/jocn.14693 | Wrong population |
| 2002 | Databases and registers | Holroyd, E. A., Bond, M. H., & Chan, H. Y. (2002). Perceptions of sex-role stereotypes, self-concept, and nursing role ideal in Chinese nursing students. *J Adv Nurs*, *37*(3), 294-303. https://doi.org/10.1046/j.1365-2648.2002.02091.x | Wrong population |
| 1981 | Databases and registers | Roberts, K. L. (1981). Perspectives on attitudes to nursing in Australian nursing students. *J Adv Nurs*, *6*(2), 117-123. https://doi.org/10.1111/j.1365-2648.1981.tb03201.x | Wrong population |
| 2009 | Databases and registers | Bolan, C., & Grainger, P. (2009). Students in the BN program--do their perceptions change? *Nurse Educ Today*, *29*(7), 775-779. https://doi.org/10.1016/j.nedt.2009.03.016 | Wrong population |
| 2004 | Databases and registers | Jinks, A. M., & Bradley, E. (2004). Angel, handmaiden, battleaxe or whore? A study which examines changes in newly recruited student nurses' attitudes to gender and nursing stereotypes. *Nurse Educ Today*, *24*(2), 121-127. https://doi.org/10.1016/j.nedt.2003.10.011 | Wrong population |
| 1992 | Databases and registers | Johnson, M. (1992). Discard nursing stereotype image. *Fla Nurse*, *40*(8), 8-9, 11. | Wrong population |
| 1995 | Databases and registers | Moule, P. (1995). Nurse learners--do nurse tutors know them? *Nurse Educ Today*, *15*(2), 125-128. https://doi.org/10.1016/s0260-6917(95)80032-8 | Wrong population |
| 1994 | Databases and registers | Okrainec, G. D. (1994). Perceptions of nursing education held by male nursing students. *West J Nurs Res*, *16*(1), 94-107. https://doi.org/10.1177/019394599401600108 | Wrong population |
| 2022 | Databases and registers | Apaydin Cirik, V., Gül, U., & Aksoy, B. (2022). The image of nursing among nursing and other healthcare professional university students: A mixed-method study. *Nurse Educ Pract*, *59*, 103293. https://doi.org/10.1016/j.nepr.2022.103293 | Wrong population |
| 1974 | Databases and registers | Brown, J. S., Swift, Y. B., & Oberman, M. L. (1974). Baccalaureate students' images of nursing: a replication. *Nurs Res*, *23*(1), 53-59. | Wrong population |
| 1993 | Databases and registers | Jinks, A. M. (1993). A study of attitudes to gender and nursing stereotypes in newly recruited student nurses. *Nurse Educ Today*, *13*(4), 287-294. https://doi.org/10.1016/0260-6917(93)90055-7 | Wrong population |
| 2024 | Databases and registers | Uzun, L. N. (2024). The effect of creative drama on nursing image: Randomized controlled study. *Nurse Educ Pract*, *77*, 103970. https://doi.org/10.1016/j.nepr.2024.103970 | Wrong population |
| 1997 | Databases and registers | Sivberg, B., & Petersson, K. (1997). Self-image, self-values and interpersonal values among newly graduated nurses. *Nurs Ethics*, *4*(5), 407-422. https://doi.org/10.1177/096973309700400507 | Wrong population |
| 1976 | Databases and registers | Denton, J. A. (1976). Attitudes toward alternative models of unions and professional associations. *Nurs Res*, *25*(3), 178-180. | Wrong population |
| 2022 | Databases and registers | Yang, L., Xu, M., Kuang, J., Zhou, K., Zhu, X., Kong, L., Qi, L., & Liu, H. (2022). Has the COVID-19 pandemic affected nursing students' career self-efficacy and professional calling? The mediating impact of professional identity. *BMC Med Educ*, *22*(1), 757. https://doi.org/10.1186/s12909-022-03833-6 | Wrong population |
| 2020 | Databases and registers | Green, G. (2020). Examining professional values among nursing students during education: A comparative study. *Nurs Forum*, *55*(4), 589-594. https://doi.org/10.1111/nuf.12474 | Wrong population |
| 1998 | Databases and registers | Ehrenfeld, M., Shmueli, M., & Henig, M. (1998). Absorption of nursing students: new immigrants in the general academic nursing programme in Israel. *J Adv Nurs*, *27*(1), 171-178. https://doi.org/10.1046/j.1365-2648.1998.00495.x | Wrong population |
| 2024 | Databases and registers | Zhu, H., Li, X., Zhang, H., Lin, X., Qu, Y., Yang, L., Ma, Q., & Zhou, C. (2024). The association between proactive personality and interprofessional learning readiness in nursing students: The chain medication effects of perceived social support and professional identity. *Nurse Educ Today*, *140*, 106266. https://doi.org/10.1016/j.nedt.2024.106266 | Wrong population |
| 2022 | Databases and registers | Ayaz-Alkaya, S., & Terzi, H. (2022). Predictors of attitudes towards nursing profession and peer caring behaviors of the nursing students: A cross-sectional study. *Nurse Educ Today*, *116*, 105467. https://doi.org/10.1016/j.nedt.2022.105467 | Wrong population |
| 2022 | Databases and registers | Lyu, F. F., Ramoo, V., & Wang, Y. X. (2022). Career maturity, psychological resilience, and professional self-concept of nursing students in China: A nationwide cross-sectional study. *J Prof Nurs*, *42*, 58-66. https://doi.org/10.1016/j.profnurs.2022.06.003 | Wrong population |
| 2024 | Databases and registers | Choi, H., & Rajaguru, V. (2024). Influencing Factors on Career Preparation Behavior of Nursing Students in the Post COVID-19 Era. *Nurs Rep*, *14*(1), 545-555. https://doi.org/10.3390/nursrep14010042 | Wrong population |
| 2023 | Databases and registers | Wang, A., Wu, F., Lin, C., Wu, M., Jia, S., Guo, Y., Zhang, W., & Huang, F. (2023). An online 5-week professional identity program for nursing student in clinical rotation practice during the first wave of COVID-19 pandemic: A two-arm randomized trial. *Nurse Educ Pract*, *68*, 103598. https://doi.org/10.1016/j.nepr.2023.103598 | Wrong population |
| 2024 | Databases and registers | Ramjan, L. M., Maneze, D., Salamonson, Y., Zugai, J., Bail, K., Liu, X. L., & Montayre, J. (2024). Undergraduate nursing students challenge misconceptions towards men in nursing: A mixed-method study. *J Adv Nurs*, *80*(4), 1638-1651. https://doi.org/10.1111/jan.15914 | Wrong population |
| 1988 | Databases and registers | Weller, L., Harrison, M., & Katz, Z. (1988). Changes in the self and professional images of student nurses. *J Adv Nurs*, *13*(2), 179-184. https://doi.org/10.1111/j.1365-2648.1988.tb01406.x | Wrong population |
| 2017 | Databases and registers | Lockeman, K. S., Appelbaum, N. P., Dow, A. W., Orr, S., Huff, T. A., Hogan, C. J., & Queen, B. A. (2017). The effect of an interprofessional simulation-based education program on perceptions and stereotypes of nursing and medical students: A quasi-experimental study. *Nurse Educ Today*, *58*, 32-37. https://doi.org/10.1016/j.nedt.2017.07.013 | Wrong population |
| 2023 | Databases and registers | Neumbe, I. M., Ssenyonga, L., Soita, D. J., Iramiot, J. S., & Nekaka, R. (2023). Attitudes and perceptions of undergraduate nursing students towards the nursing profession. *PLoS One*, *18*(7), e0280700. https://doi.org/10.1371/journal.pone.0280700 | Wrong population |
| 2023 | Databases and registers | Allen, L., Cooper, S., & Missen, K. (2023). Perceptions of being a registered nurse (PRN): development and validation of a survey tool. *BMC Nurs*, *22*(1), 159. https://doi.org/10.1186/s12912-023-01324-7 | Wrong population |
| 2023 | Databases and registers | Alotaibi, K. A., Balay-Odao, E. M., Cruz, J. P., Alotaibi, J. S., Bajet, J. B., Alquwez, N., Mesde, J., Almansour, A. M., Almoghairi, A., & Albougami, A. S. (2023). Influence of the Psychological Effect of COVID-19 on Saudi Student Nurses' Attitudes toward Nursing and Their Future Career Plans. *J Pers Med*, *13*(4). https://doi.org/10.3390/jpm13040672 | Wrong population |
| 2021 | Databases and registers | Alshammari, F. F., Grande, R. A. N., & Berdida, D. J. E. (2021). The correlates of social determinants to ethico-moral values on professional development of Saudi nursing students. *J Taibah Univ Med Sci*, *16*(2), 240-246. https://doi.org/10.1016/j.jtumed.2020.11.002 | Wrong population |
| 2024 | Databases and registers | Sacgaca, L., An, E. P., Alqarni, A. S., Pangket, P., Alshammari, S. A., Ramadan, M. Y., Alonezei, A. K., Alamoudi, F. A., Mohammed, I. H. A., Cabansag, D., & Benjamin, L. S. (2024). Sociocultural and perceived public image of nurses among nursing students: the mediating role of self-concept. *BMC Nurs*, *23*(1), 298. https://doi.org/10.1186/s12912-024-01957-2 | Wrong population |
| 2017 | Databases and registers | Čukljek, S., Jureša, V., Bile, C. G., & Režek, B. (2017). Changes in nursing students' attitudes towards nursing during undergraduate study [Article]. *Acta Clinica Croatica*, *56*(1), 36-43. https://doi.org/10.20471/acc.2017.56.01.06 | Wrong population |
| 2019 | Databases and registers | Haghighat, S., Borhani, F., Ranjbar, H., & Naseri, P. (2019). Evaluating the formation of professional identity in Iranian nursing students after implementation of a new curriculum. *Journal of Nursing & Midwifery Sciences*, *6*(3), 138-143. https://doi.org/10.4103/JNMS.JNMS_1_19 | Wrong population |
| 2022 | Databases and registers | Emire, M. S., Haile, T. G., & Tesu, M. (2022). Assessment of Students' Attitudes Towards the Nursing Profession at Wolkite University, Ethiopia, 2021. *Nursing: Research & Reviews*, *12*, 159-168. https://doi.org/10.2147/NRR.S371365 | Wrong population |
| 1998 | Databases and registers | Huffstutler, S. Y., Stevenson, S. S., Mullins, I. L., Hackett, D. A., & Lambert, A. W. (1998). The public's image of nursing as described to baccalaureate prenursing students. *Journal of Professional Nursing*, *14*(1), 7-13. https://doi.org/10.1016/s8755-7223(98)80007-0 | Wrong population |
| 2007 | Databases and registers | Wallace, C. B. (2007). *Nursing students' perceptions of the public image of nursing* (Publication Number Ph.D.) Capella University]. Cinahl. https://libproxy1.nus.edu.sg/login?url=https://search.ebscohost.com/login.aspx?direct=true&db=ccm&AN=109849566&site=ehost-live | Wrong population |
| 2009 | Databases and registers | Lovan, S. R. (2009). *Comparing perceptions of the nursing profession among associate and baccalaureate nursing students and registered nurses* (Publication Number Ph.D.) University of Louisville]. Cinahl. https://libproxy1.nus.edu.sg/login?url=https://search.ebscohost.com/login.aspx?direct=true&db=ccm&AN=109853316&site=ehost-live | Wrong population |
| 2010 | Databases and registers | Bartfay, W. J., Bartfay, E., Clow, K. A., & Wu, T. (2010). Attitudes and perceptions towards men in nursing education. *Internet Journal of Allied Health Sciences & Practice*, *8*(2), 7p-7p. https://libproxy1.nus.edu.sg/login?url=https://search.ebscohost.com/login.aspx?direct=true&db=ccm&AN=105211514&site=ehost-live | Wrong population |
| 1991 | Databases and registers | May, F. E., Champion, V., & Austin, J. K. (1991). Public values and beliefs toward nursing as a career. *J Nurs Educ*, *30*(7), 303-310. https://doi.org/10.3928/0148-4834-19910901-06 | Wrong population |
| 2024 | Databases and registers | Apaydin Cirik, V., Aksoy, B., & Bektaş, M. (2024). Development of Adolescents' perceptions of the nursing image scale and evaluation of its psychometric properties: A methodological study. *J Pediatr Nurs*, *75*, e119-e127. https://doi.org/10.1016/j.pedn.2024.01.002 | Psychometric properties testing |
| 2005 | Databases and registers | Woodgate, R. L. (2005). A different way of being: adolescents' experiences with cancer. *Cancer Nurs*, *28*(1), 8-15. https://doi.org/10.1097/00002820-200501000-00002 | Psychometric properties testing |
| 2023 | Databases and registers | Glerean, N., Talman, K., Glerean, E., Hupli, M., & Haavisto, E. (2023). Development and psychometric testing of the perception of nursing profession instrument. *J Adv Nurs*, *79*(10), 4074-4087. https://doi.org/10.1111/jan.15726 | Psychometric properties testing |
| 2021 | Databases and registers | Sharma, S. K., & Mudgal, S. K. (2021). Development and validation of a scale to measure attitude of people toward men in nursing profession. *J Educ Health Promot*, *10*, 54. https://doi.org/10.4103/jehp.jehp_530_20 | Psychometric properties testing |
| 1985 | Databases and registers | Domínguez Alcón, C. (1985). [Image of the nurse in society]. *Rev Enferm*, *8*(83), 66-72. (Imagen de la enfermera en la sociedad.) | Non-English/ Non-Mandarin |
| 1982 | Databases and registers | Mizutani, M., & Hori, N. (1982). [Changing attitudes of nursing students before and after clinical training-with special reference to their attitude to the training, image of the nurse, professional knowledge, and technics]. *Kango Tenbo*, *7*(6), 560-569. | Non-English/ Non-Mandarin |
| 2024 | Databases and registers | Viottini, E., Ferrero, A., Acquaro, J., Bulfone, G., Condemi, F., D'Accolti, D., Massimi, A., Mattiussi, E., Sturaro, R., Conti, A., & Dimonte, V. (2024). [Investing in healthcare professionals. The motivation for enrollment in bachelor nursing courses: results from a pilot study]. *Assist Inferm Ric*, *43*(1), 6-15. https://doi.org/10.1702/4250.42266 | Non-English/ Non-Mandarin |
| 2016 | Databases and registers | Albar, M. J., & Sivianes-Fernández, M. (2016). [Perception of professional identity in nursing amongst undergraduate students]. *Enferm Clin*, *26*(3), 194-198. https://doi.org/10.1016/j.enfcli.2015.10.006 | Non-English/ Non-Mandarin |
| 2017 | Databases and registers | Ashkenazi, L., Livshiz-Riven, I., Romem, P., & Grinstein-Cohen, O. (2017). Male Nurses in Israel: Barriers, Motivation, and How They Are Perceived by Nursing Students. *J Prof Nurs*, *33*(2), 162-169. https://doi.org/10.1016/j.profnurs.2016.08.001 | Does not directly examine nursing image |
| 2005 | Databases and registers | Jrasat, M., Samawi, O., & Wilson, C. (2005). Beliefs, attitudes and perceived practice among newly enrolled students at the Jordanian Ministry of Health nursing colleges and institutes in 2003. *Educ Health (Abingdon)*, *18*(2), 145-156. https://doi.org/10.1080/13576280500148767 | Does not directly examine nursing image |
| 1991 | Databases and registers | McCarty, P. (1991). Image campaign boosts student enrollment. *Am Nurse*, *23*(9), 1, 24. | Does not directly examine nursing image |
| 2016 | Databases and registers | Yilmaz, A. A., Ilce, A., Can Cicek, S., Yuzden, G. E., & Yigit, U. (2016). The effect of a career activity on the students' perception of the nursing profession and their career plan: A single-group experimental study. *Nurse Educ Today*, *39*, 176-180. https://doi.org/10.1016/j.nedt.2016.02.001 | Does not directly examine nursing image |
| 2004 | Databases and registers | Al-Omar, B. A. (2004). Knowledge, attitudes and intention of high school students towards the nursing profession in Riyadh city, Saudi Arabia. *Saudi Med J*, *25*(2), 150-155. | Does not directly examine nursing image |
| 2016 | Databases and registers | Ben Natan, M. (2016). Interest in nursing among academic degree holders in Israel: A cross-sectional quantitative study. *Nurse Educ Today*, *38*, 150-153. https://doi.org/10.1016/j.nedt.2015.11.025 | Does not directly examine nursing image |
| 1976 | Databases and registers | Tetreault, A. I. (1976). Selected factors associated with professional attitude of baccalaureate nursing students. *Nurs Res*, *25*(1), 49-53. | Does not directly examine nursing image |
| 1998 | Databases and registers | Sivberg, B. (1998). Self-perception and value system as possible predictors of stress. *Nurs Ethics*, *5*(2), 103-121. https://doi.org/10.1177/096973309800500203 | Does not directly examine nursing image |
| 2013 | Databases and registers | Myers, L. H. (2013). Partnering to provide a summer institute of nursing. *J Nurs Educ*, *52*(12), 701-704. https://doi.org/10.3928/01484834-20131121-02 | Does not directly examine nursing image |
| 1958 | Databases and registers | Meyer, G. R. (1958). The attitude of student nurses toward patient contact and their images of and preferences for four nursing specialties. *Nurs Res*, *7*(3), 126-130. https://doi.org/10.1097/00006199-195810000-00006 | Does not directly examine nursing image |
| 1981 | Databases and registers | Kalisch, P. A., & Kalisch, B. J. (1981). The image of psychiatric nurses in motion pictures. *Perspect Psychiatr Care*, *19*(3-4), 116-129. https://doi.org/10.1111/j.1744-6163.1981.tb00125.x | Does not directly examine nursing image |
| 1988 | Databases and registers | Dobbs, K. K. (1988). The senior preceptorship as a method for anticipatory socialization of baccalaureate nursing students. *J Nurs Educ*, *27*(4), 167-171. https://doi.org/10.3928/0148-4834-19880401-07 | Does not directly examine nursing image |
| 2000 | Databases and registers | Kikwilu, E. N., Mugonzibwa, E. A., Rugarabamu, P. G., & Ntabaye, M. K. (2000). Tanzanian high school students' attitude towards five university professional courses. *East Afr Med J*, *77*(3), 143-146. https://doi.org/10.4314/eamj.v77i3.46610 | Does not directly examine nursing image |
| 2024 | Databases and registers | Wang, R., Yu, H., Han, X., Yang, Y., Chen, D., Niu, Q., Liu, Y., Zhou, M., Zhu, X., & Zhang, Y. (2024). Identifying the personal characteristics of decent work perception for nursing students in China using latent profile analysis. *BMC Med Educ*, *24*(1), 315. https://doi.org/10.1186/s12909-024-05206-7 | Does not directly examine nursing image |
| 2018 | Databases and registers | Merati, N., Murphy-Buske, A., Alfaro, P., Larouche, S., Noel, G., & Ventura, N. (2018). The effects of an anatomy near-peer learning activity on interprofessional stereotypes: A mixed methods study [Conference Abstract]. *FASEB Journal*, *32*(1). https://www.embase.com/search/results?subaction=viewrecord&id=L622541654&from=export | Does not directly examine nursing image |
| 2008 | Databases and registers | Donelan, K., Buerhaus, P., DesRoches, C., Dittus, R., & Dutwin, D. (2008). Public perceptions of nursing careers: the influence of the media and nursing shortages. *Nursing Economic$*, *26*(3), 143-165. https://libproxy1.nus.edu.sg/login?url=https://search.ebscohost.com/login.aspx?direct=true&db=ccm&AN=105787413&site=ehost-live | Does not directly examine nursing image |
| 2019 | Databases and registers | Sullivan, K., Guzman, A. S., & Ghaffari, D. (2019). The Relationship Between Social Identity, Stereotype Threat, and Academic Success Among Prenursing Students. *Nursing Education Perspectives (Wolters Kluwer Health)*, *40*(6), E16-E18. https://doi.org/10.1097/01.NEP.0000000000000578 | Does not directly examine nursing image |
| 2014 | Databases and registers | Clow, K. A., Ricciardelli, R., & Bartfay, W. J. (2014). Attitudes and Stereotypes of Male and Female Nurses: The Influence of Social Roles and Ambivalent Sexism. *Canadian Journal of Behavioural Science*, *46*(3), 446-455. https://doi.org/10.1037/a0034248 | Does not directly examine nursing image |
| 1988 | Databases and registers | Hitchen, J. M., & Brown, R. A. (1988). A curriculum design for collaboration in basic nurse education. *Nurse Education Today*, *8*(6), 341-346. https://libproxy1.nus.edu.sg/login?url=https://search.ebscohost.com/login.aspx?direct=true&db=ccm&AN=107529250&site=ehost-live | Does not directly examine nursing image |
| 1966 | Databases and registers | Lande, S. (1966). Nursing career perceptions among high school students. *Nursing Research*, *15*, 337-337. https://doi.org/10.1097/00006199-196615040-00009 | Does not directly examine nursing image |
| 2010 | Databases and registers | Shaw, K., & Timmons, S. (2010). Exploring how nursing uniforms influence self image and professional identity. *Nurs Times*, *106*(10), 21-23. | Excluded study design |
| 2009 | Databases and registers | Dunlap, J. M., & Olshansky, E. (2009). Why not become an M.D.? A perspective from a student nurse on the profession's image. *J Prof Nurs*, *25*(2), 65-66. https://doi.org/10.1016/j.profnurs.2009.02.002 | Excluded study design |
| 2012 | Databases and registers | Cook, R. (2012). Is nursing's image going through a professional adolescence? *Nurs Times*, *108*(26), 7. | Excluded study design |
| 1980 | Databases and registers | Baer, C. M. (1980). Groundwork for recruitment into nursing: Images of nursing among adolescents. https://www.embase.com/search/results?subaction=viewrecord&id=L12210424&from=export | Excluded study design |
| 2001 | Databases and registers | Rasmussen, E. (2001). The adventure begins: youth recruitment video promotes modern image of nursing. *NurseWeek (15475131)*, *6*(5), 20-21. https://libproxy1.nus.edu.sg/login?url=https://search.ebscohost.com/login.aspx?direct=true&db=ccm&AN=107062007&site=ehost-live | Excluded study design |

**Table S5** *Data Extraction Table*

| **Author (Year), Country** | **Design** | **Population (n)** | **Aim** | **Data source / instrument** | **Theory based** | **Key review-relevant findings** | **Main factor(s) identified** |
| --- | --- | --- | --- | --- | --- | --- | --- |
| **Al-Kandari & Lew (2005)**  *Kuwait* | Quantitative descriptive design | All-female high school students (n=289) | To explore Kuwaiti high school students’ perceptions of nursing as a profession and factors affecting their choice of nursing as a career. | **Data source:** Questionnaire  **Instrument:** A self-administered questionnaire was developed in Arabic by the first author | Not reported | **Nursing-image findings:**   - Students knew nursing tasks, cultural and gender barriers shaped perceptions.   **Career-related findings:**   - Only 19% considered it as a career. | **Individual level**  • Gender Stereotypes  • Knowledge of the Profession  **Interpersonal level**  • Exposure to Nursing Professionals  **Societal level**  • Sociocultural Norms |
| **Andrews (2005)**  *United States* | Cross-sectional design | High school boys (n=222) | To explore the influence of societal expectations and demographic variables on high school boys' perceptions of nursing as a career choice, including factors such as parental, teacher, and peer influences. | **Data source:** Questionnaire  **Instrument:** Adapted tool from Attitudes, Values, and Beliefs Questionnaire (May et al., 1988) | Gottfredson's Circumscription and Compromise Theory of career development (1981) | **Nursing-image findings:**   - Boys viewed nursing as respectable but female-typed; reduced gender-role beliefs and personal exposure improved perceptions, while demographics were not influential. | **Individual level**  • Gender Stereotypes  **Interpersonal level**  • Peer Influence  • Exposure to Nursing Professionals |
| **Barkley & Kohler (1992)**  *USA* | Quantitative descriptive design | Male high school students (n=126) | To investigate male high school students' perceptions of nursing and the factors that deter them from considering it as a career | **Data source:** Questionnaire  **Instrument:** Career Questionnaire (Kohler & Edwards, 1990) | Not reported | **Nursing-image findings:**   - Male students viewed nurses positively but female stereotypes and nurturing-role assumptions contributed to a gendered nursing image. | **Individual level**  • Gender Stereotypes |
| **Ben Natan et al. (2024)**  *Israel* | Cross-sectional study | Young Muslim Arab women (n=200) | To explore the influence of nurse uniforms, the COVID-19 pandemic, and societal perceptions of the nursing profession on career choices among young Muslim Arab women. | **Data source:** Questionnaire  **Instrument:** Nursing Image Scale (Porter & Porter, 1991) | Not reported | **Nursing-image findings:**   - COVID-19 raising visibility of nurses, but persistent societal stereotypes of nursing as low-status and female-dominant. | **Societal level**  • Media Portrayals  • Sociocultural Norms |
| **Cohen et al. (2004)**  *USA* | Descriptive comparative study | Middle school students (n=301) | To compare middle school students' perceptions of an ideal career with their perceptions of nursing | **Data source:** Questionnaire  **Instrument:** Adapted instrument by May, Champion & Austin (1991) | Not reported | **Nursing-image findings:**   - Nursing was seen as less autonomous, although exposure to nurses improved perceptions   **Career-related findings:**   - Nursing was seen as less financially rewarding than other careers | **Individual level**  • Knowledge of the Profession  • Perceived Work Attributes  **Interpersonal level**  • Exposure to Nursing Professionals |
| **Degazon & Shaw (2007)**  *USA* | Quantitative descriptive design | Urban high school students (n=114) | To examine the perceptions of urban high school students towards nursing as a potential career | **Data source:** Questionnaire  **Instrument:** Attitudes, Values, and Beliefs Questionnaire (May et al., 1988) | Not reported | **Nursing-image findings:**   - Positive role models and healthcare exposure improved views - low pay, long hours, and female stereotypes contributed to a less favorable image of nursing. | **Individual level**  • Gender Stereotypes  • Perceived Work Attributes  **Interpersonal level**  • Exposure to Nursing Professionals |
| **Degazon et al. (2015)**  *USA and Israel* | Quantitative descriptive design | High school students (n=330) | To compare ethnic minority high school students' perceptions of nursing as a career in the USA and Israel, and how these perceptions compare to their views on an ideal career | **Data source:** Questionnaire  **Instrument:** Attitudes, Values, and Beliefs Questionnaire (May et al., 1988) | Not reported | **Nursing-image findings:**   - USA students viewed nursing more positively than Israeli students - Both groups saw it as laborious, lacking leadership opportunities, and offering less financial reward than other professions. | **Individual level**  • Perceived Work Attributes  **Societal level**  • Sociocultural Norms |
| **Drenkard et al. (2002)**  *USA* | Pretest-posttest intervention design | Middle school students (n=20) | To promote a positive image of nursing and encourage students to consider nursing as a career through hands-on experiences in a nursing summer camp. | **Data source:** Pre- and post-camp surveys and daily evaluations  **Instrument:** Adapted survey from Grossman’s study (1989) | Not reported | **Nursing-image findings:**   - The camp successfully improved students’ perceptions of nursing, with significant responsibilities beyond caregiving.   **Career-related findings:**   - Participants viewing the profession as one with opportunities for career advancement | **Societal level**  • Targeted Recruitment Interventions |
| **Droes et al. (1993)**  *USA* | Pretest-posttest control group study | High school biology students (n=451) | To evaluate the effects of a participative intervention on students’ image and valuation of nursing. | **Data source:** Dialogue and questionnaires  **Instrument:** Nursing Image Questionnaire and Nursing Valuation tool by faculty researchers | Diffusion change theory and Social Intervention Framework | **Nursing-image findings:**   - The intervention was effective in broadening students’ perception of nursing, particularly in terms of autonomy, scientific knowledge, and caring. | **Societal level**  • Targeted Recruitment Interventions |
| **Elmorshedy et al. (2020)**  *Saudi Arabia* | Cross-sectional study | Students age less than 20 (n=502) | To explore perceptions and socio-cultural barriers related to the nursing profession in Saudi Arabia. | **Data source:** Questionnaire  **Instrument:** A self-administered questionnaire | Not reported | **Nursing-image findings:**   - Awareness of nursing was low among pre-nursing students, and perceived barriers did not differ by age group. | **Individual level**  • Knowledge of the Profession |
| **Foong et al. (1999)**  *Hong Kong* | Qualitative descriptive study | High school students (n=19) | To explore the perceptions of high school students in Hong Kong regarding the nursing profession, their understanding of nurses’ roles. | **Data source:** Structured interview  **Instrument:** Structured interview guides | Not reported | **Nursing-image findings:**   - Students had limited understanding of nursing and viewed it as low-status and subordinate - family and television often reinforced stereotypes. | **Individual level**  • Knowledge of the Profession  • Perceived Work Attributes  **Interpersonal level**  • Family Influence  • Peer Influence  **Societal level**  • Media Portrayals |
| **Hemsley-Brown & Foskett (1999)**  *United Kingdom* | Mixed-methods study | Young people (n=410) | To examine and analyze young people's perceptions of nursing at key stages in education decision-making | **Data source:** Questionnaires and focus group discussions  **Instrument:** Open-ended questionnaires. | Not reported | **Nursing-image findings:**   - Nursing was seen as caring but lower in status and ambition, and strong feminine associations.   **Career-related findings:**   - Nursing was seen with limited career progression | **Individual level**  • Gender Stereotypes  • Perceived Work Attributes  • Intrinsic Motivation |
| **Joung & Shin (2018)**  *South Korea* | Cross-sectional study | High school girls (n=30) | To identify and categorize how high school girls perceive nurses' image and analyze these perceptions to inform educational strategies for future nursing students | **Data source:** Questionnaire  **Instrument:** Q methodology, with 29 Q statements scored on a 7-point scale | Not reported | **Nursing-image findings:**   - Hospital exposure, family members in nursing, and TV dramas shaped perceptions. - Students with family members in nursing were more likely to hold positive perceptions. - Overall, nurses were often perceived as secondary to doctors and seen as playing ancillary rather than scientific or leadership roles. | **Interpersonal level**  • Family Influence  • Exposure to Nursing Professionals  **Societal level**  • Media Portrayals  • Sociocultural Norms |
| **Kohler & Edwards (1990)**  *USA* | Descriptive quantitative study | High school students (n=306) | To investigate high school students' beliefs and perceptions about nursing, including educational requirements, working conditions, salary, and status | **Data source:** Questionnaire  **Instrument:** Investigator-designed questionnaire | Not reported | **Nursing-image findings:**   - Nursing seen as demanding, low-status, and poorly rewarded. - Many believed nurses had low social status, often equating them with secretaries, and thought plumbers earned more. - Personal contact with nurses and direct hospital encounters.   **Career-related findings:**   - Few students (8.6%) considered nursing | **Individual level**  • Knowledge of the Profession  **Interpersonal level**  • Exposure to Nursing Professionals  **Societal level**  • Sociocultural Norms |
| **Luchesi et al. (2009)**  *Brazil* | Quantitative-descriptive pilot study | High school students (n=46) | To analyze secondary-level students' perceptions of the nursing profession and develop hypotheses about the presence or absence of negative stereotypes towards nurses | **Data source:** Questionnaire  **Instrument:** Social status and salary ranking of profession including nursing, and Nursing-specific stereotypes and contact with nursing professionals | Not reported | **Nursing-image findings:**   - Students recognized caring qualities but underestimated nurses’ education; - Personal contact improved views, while gender stereotypes discouraged males. | **Individual level**  • Gender Stereotypes  • Knowledge of the Profession  **Interpersonal level**  • Exposure to Nursing Professionals |
| **Marriner-Tomey et al. (1990)**  *USA* | Descriptive comparative study | Sophomore high school students (n=341) | To compare sophomore high school students' perceptions of an ideal career with their perceptions of nursing as a career | **Data source:** Questionnaire  **Instrument:** Modified version of the questionnaire developed by May, Austin & Champion (1988) | Not reported | **Nursing-image findings:**   - Students valued nursing as a profession characterized by hard work and care but did not see it as meeting ideals of autonomy and high financial reward. - Familiarity with nursing through relatives or personal experience improved perceptions. | **Individual level**  • Gender Stereotypes  • Knowledge of the Profession  • Perceived Work Attributes  **Interpersonal level**  • Exposure to Nursing Professionals |
| **Matutina (2012)**  *USA* | Cluster randomized controlled design | Middle school students (n=80) | To evaluate how educational interventions can improve the perception of nursing, specifically its image, among middle school students | **Data source:** Questionnaire  **Instrument:** Indiana instrument | Social cognitive theory | **Nursing-image findings:**   - Students initially saw nursing as subordinate, physically demanding, and low paid - The intervention improved views of skill, and knowledge.   **Career-related findings:**   - Intervention improved views of career breadth. | **Individual level**  • Knowledge of the Profession  **Societal level**  • Sociocultural Norms  • Targeted Recruitment Interventions |
| **Maymoun & Sohail (2020)**  *United Arab Emirates* | Cross-sectional descriptive design | Female high school students (n=157) | To examine the relationship between attitude, knowledge, and intention to pursue a nursing career | **Data source:** Questionnaire  **Instrument:** Measure attitudinal factors through identifying eight items from Kim et al. (2013) study | Theory of reasoned action | **Nursing-image findings:**   - Relatives or friends in nursing and helping-oriented motives supported positive perceptions   **Career-related findings:**   - Job security and varied opportunities also increased appeal despite some negative societal views. | **Individual level**  • Perceived Work Attributes  • Intrinsic Motivation  **Interpersonal level**  • Family Influence  **Societal level**  • Sociocultural Norms |
| **May et al. (1991)**  *USA* | Quantitative descriptive design | High school-age students (n=330) | To compare and determine the attitudes and perceptions of “ideal career” and “nursing as a career” | **Data source:** Questionnaire  **Instrument:** Self-developed 17-item survey scales | Not reported | **Nursing-image findings:**   - Nursing was viewed as caring, respectful and using intellectual ability, but less favorable for leadership. - High school students and their “enablers” (e.g. parents) tended to view nursing as less favorable.   **Career-related findings:**   - Less favorable than an ideal career for power and pay. | **Individual level**  • Knowledge of the Profession  • Perceived Work Attributes  **Interpersonal level**  • Family Influence |
| **Mohsen et al. (2022)**  *Israel* | Cross-sectional | High school students (n=138) | To examine the relationship between the public image of nursing and the intention to choose it as a career | **Data source:** Questionnaire  **Instrument:** Structured questionnaire | Not reported | **Nursing-image findings:**   - A positive public image, higher paternal education, and prior exposure to nurses were associated with more favorable perceptions of nursing. | **Interpersonal level**  • Family Influence  • Exposure to Nursing Professionals |
| **Murray (2002)**  *USA* | Qualitative descriptive study | High school students (n=74) | To explore how high school students interpret the image of nursing in American movies and television, and whether these perceptions influence their career decision to choose or not choose nursing. | **Data source:** Nine focus groups, supported by qualitative computer software (NUD*IST) for thematic analysis.  **Instrument:** Written questionnaires | Not reported | **Nursing-image findings:**   - Media strongly shaped perceptions, portraying nursing as caring but also subordinate and stereotyped, alongside wider social pressures. - Media portrayals were as influential as personal experiences in shaping students’ image of nursing, alongside societal pressure, and recruitment strategies | **Individual level**  • Knowledge of the Profession  • Intrinsic Motivation  **Interpersonal level**  • Peer Influence  • Exposure to Nursing Professionals  **Societal level**  • Media Portrayals |
| **Palazzo & Erickson (2022)**  *USA* | Quantitative descriptive study | High school students from a large Pacific Northwest school district (n=820) | To understand male high school students' perceptions of nursing as a professional career choice | **Data source:** Questionnaire  **Instrument:** 32-item descriptive survey | Theory of planned behavior | **Nursing-image findings:**   - Self-identified female students and self-identified gay and/or bisexual male students had more positive perceptions of the nursing profession compared with other self-identified male students. | **Individual level**  • Gender Stereotypes |
| **Öncü et al. (2022)**  *Turkey* | Cross-sectional study | High school students (n=745) | To evaluate the nursing image and social status characteristics on young people’s views on nursing and its social image in Turkey. | **Data source:** Questionnaire  **Instrument:** Nursing Image Scale (Dost & Bahçecik, 2015) | Not reported | **Nursing-image findings:**   - Nursing’s image was constrained by historical and sociocultural stereotypes; - Female students from lower-income backgrounds viewed nursing more positively as stable, care-oriented work, whereas higher-income male students regarded it as low-status and gender-segregated; personal connections improved views, while COVID-related media had limited impact. | **Individual level**  • Gender Stereotypes  • Knowledge of the Profession  **Interpersonal level**  • Exposure to Nursing Professionals  **Societal level**  • Media Portrayals  • Sociocultural Norms |
| **Rambur et al. (2011)**  *USA* | Quantitative descriptive design | Young adults (n=116) | To understand gender differences in young adults’ perceptions of an ideal career and their views of nursing | **Data source:** Survey  **Instrument:** Instrument developed by May et al. (1991) | Not reported | **Nursing-image findings:**   - Women prioritized respect and appreciation, men prioritized challenge and pay; nursing was viewed as lower in status, particularly by male participants. | **Individual level**  • Gender Stereotypes |
| **Raymond et al. (2018)**  *Australia* | Mixed-methods pre-post intervention study | Secondary school students (n=71) | To identify a method of attracting final year secondary school students to an undergraduate nursing degree at a rural University | **Data source:** Semi structured questionnaires  **Instrument:** Self-developed survey | Not reported | **Nursing-image findings:**   - Students’ understanding of the nursing role was limited, with many relying on family, friends, and media to form their views. - After the intervention, students reported more favorable views of nursing, with improved understanding of the nursing role and its professional opportunities. | **Individual level**  • Knowledge of the Profession  **Interpersonal level**  • Family Influence  • Peer Influence  **Societal level**  • Targeted Recruitment Interventions |
| **Reiskin & Haussler (1994)**  *USA* | Cross-sectional survey | Junior year high school students (n=276) | To examine how students from different cultural and racial groups perceive nursing and how these perceptions compare to their views of an ideal career | **Data source:** Questionnaire  **Instrument:** Instrument developed by May, Austin, and Champion's (1988) questionnaire | Not reported | **Nursing-image findings:**   - Students saw nurses as caring and hardworking but not as leaders or well-paid professionals - Perceptions varied across cultural and racial group, with African American and Latino female students holding more positive views of nursing than other groups, particularly Asian female students. | **Individual level**  • Knowledge of the Profession  **Societal level**  • Sociocultural Norms |
| **Rossiter et al. (1998)**  *Australia* | Descriptive quantitative study | Non-English speaking background (NESB) high school students (n=162) | To examine NESB high school students' career preferences in relation to nursing and whether nursing is perceived differently across Korean, Lebanese, Vietnamese, and mainland Chinese students | **Data source:** Questionnaire  **Instrument:** 56-item questionnaire designed by the researchers | Leininger’s Cultural Care Diversity and Universality Theory (1991) | **Nursing-image findings:**   - Korean students had the lowest attitude scores, while Vietnamese students showed a relatively higher inclination towards nursing. - Knowledge gaps were common, and many students still viewed nurses primarily as assistants to doctors. | **Individual level**  • Knowledge of the Profession  **Societal level**  • Sociocultural Norms |
| **Rossiter et al. (1999)**  *Hong Kong* | Quantitative descriptive design | High school students (n=375) | To examine high school students’ attitudes towards the nursing profession and to identify the contributing factors affecting shortage of nurses within the context of Hong Kong | **Data source:** Questionnaire  **Instrument:** 35-item questionnaire assessing knowledge, attitudes, and intention towards nursing | Not reported | **Nursing-image findings:**   - Students understood nursing reasonably well but some continued to hold less favorable perceptions. - Acquaintance with a nurse slightly improved attitudes and intention. | **Individual level**  • Knowledge of the Profession  • Perceived Work Attributes  **Interpersonal level**  • Family Influence  • Exposure to Nursing Professionals |
| **Rubbi et al. (2019)**  *Italy* | Cross-sectional observational study | High school students (n=490) | To investigate the impact of orientation on high school students' perceptions of nursing and their likelihood to choose it as a career. | **Data source:** Questionnaire  **Instrument:** 30-item Nursing Attitude Questionnaire (NAQ) | Not reported | **Nursing-image findings:**   - Students who attended orientation had higher nursing-image scores, particularly for professional roles, professional values, and stereotypes, than those who did not attend. - Female students had more positive perceptions of nursing than male students. | **Interpersonal level**  • Family Influence  • Exposure to Nursing Professionals  **Societal level**  • Targeted Recruitment Interventions |
| **Tang et al. (1999)**  *Australia* | Cross-sectional survey | High school Year 11 and 12 students (n=789) | To examine the perceptions of nursing as a career choice among students from multicultural backgrounds and explore their views on the ideal career compared to nursing. | **Data source:** Questionnaire  **Instrument:** Self-developed questionnaire | Not reported | **Nursing-image findings:**   - Students linked nursing with helping and hard work more than leadership or decision-making - Perceptions varied across language groups - Significant differences were observed between language groups in views on leadership, social recognition, and knowledge. | **Individual level**  • Knowledge of the Profession  • Perceived Work Attributes  **Societal level**  • Sociocultural Norms |
| **Tawash & Cowman (2018)**  *Bahrain* | Mixed-methods study (one-group pretest-posttest) | High school students (n=685) | To identify factors influencing Arabic high school students’ choice of nursing and propose strategic interventions to promote nursing in the Arab region. | **Data source:** Questionnaire, focus group, semi-structured interviews  **Instrument:** Adapted questionnaire from Anderson (1992), based on Self-Determination Theory | Deci & Ryan's Self-Determination Theory (1985) | **Nursing-image findings:**   - Cultural barriers, such as gender roles and societal expectations, continued to shape less favorable perceptions of the profession. - Recruitment campaigns were reported to improve perceptions of nursing image.   **Career-related findings:**   - Nursing was perceived positively as a secure job | **Individual level**  • Gender Stereotypes  • Knowledge of the Profession  **Societal level**  • Sociocultural Norms  • Targeted Recruitment Interventions |
| **Zamanzadeh et al. (2023)**  *Iran* | A qualitative study with a conventional content analysis design. | Nursing school applicants (n=19) | To describe the perceptions of nursing applicants about their chosen profession and to explore the factors which influenced their understanding. | **Data source:** Semi-structured interviews  **Instrument:** Semi-structured interview guide | Not reported | **Nursing-image findings:**   - Applicants viewed nursing as feminine, physician-directed, and limited in autonomy and advancement, despite job security - Perceptions were shaped by media portrayals, advice from career counsellors, personal factors (such as prior interest in care or hospital experience), and the opinions of family and friends. | **Individual level**  • Gender Stereotypes  • Knowledge of the Profession  • Perceived Work Attributes  • Intrinsic Motivation  **Interpersonal level**  • Family Influence  • Peer Influence  • Exposure to Nursing Professionals  **Societal level**  • Media Portrayals |

**Table S6** *Geographic distribution of included studies*

| **Region** | **Country** | **Number of studies, k(%)** |
| --- | --- | --- |
| North America | United States | 15 (46.9%) |
| Middle East | Israel | 3 (9.4%) |
|  | Kuwait | 1 (3.1%) |
|  | Saudi Arabia | 1 (3.1%) |
|  | United Arab Emirates | 1 (3.1%) |
|  | Bahrain | 1 (3.1%) |
|  | Turkey | 1 (3.1%)* |
| Asia | Hong Kong | 2 (6.3%) |
|  | South Korea | 1 (3.1%) |
|  | Iran | 1 (3.1%) |
| Europe | United Kingdom | 1 (3.1%) |
|  | Italy | 1 (3.1%) |
| South America | Brazil | 1 (3.1%) |
| Oceania | Australia | 3 (9.4%) |

Note. *Percentages are based on 32 included studies and sum to more than 100% because Degazon et al. (2015) was conducted across two countries (United States and Israel) and is counted under both.

**Table S7** *Summary of quantitative findings*

| **Study** | **Image-related descriptive statistics** | **Statistical comparison / standardized p-value** | **Effect size / estimate** |
| --- | --- | --- | --- |
| Al-Kandari & Lew (2005) | Broad knowledge required: 92%; physically exhausting: 91%; stimulates mental work: 88%; respected in Kuwaiti society: 85%; safe work environment: 77%; challenging profession: 76%; advanced technology: 75%; adequate salary: 60%; unpleasant duties: 50%; able to choose working hours/vacations: 45%. | No inferential comparison directly examining nursing image constructs was reported. | Descriptive percentages only; effect size not reported. |
| Andrews (2005) | Image dimensions assessed: prestige, gender-type, and work activity. | Nursing acceptability for self: Wilks’ Λ = 0.957, canonical r = 0.207, p = 0.023. Nursing acceptability for a male peer: Wilks’ Λ = 0.809, canonical r = 0.438, p < 0.001. Significant predictors: prestige, p = 0.008; prestige and gender-type for male peer, p < 0.001 and p = 0.001, respectively. | Canonical r = 0.207 and 0.438. Effect size not reported. |
| Barkley & Kohler (1992) | Nursing is a profession: 86%; kind/compassionate: 78%; make important decisions: 74%; high standards of behaviour: 72%; good public image: 71%; stimulating/challenging: 69%; unpleasant tasks: 83%; stressful work: 78%; work too hard: 57%; rejected “only women should be nurses”: 73%; rejected “male nurses are wimps”: 77%. | No inferential statistical comparison was reported. | Descriptive percentages only; effect size not applicable. |
| Ben Natan et al. (2024) | Image-related variables assessed: image of nurses, image of the nursing profession, nurse uniforms, and COVID-19 effect. | Correlations with nursing-related consideration: image of nurses, r = 0.187, p < 0.01; image of nursing profession, r = 0.511, p < 0.01; blue uniform, r = 0.295, p < 0.01; multicoloured uniform, r = 0.168, p < 0.05; white uniform, r = 0.351, p < 0.01; COVID-19 effect, r = 0.273, p < 0.01. Regression: image of nursing profession, β = 0.489, p < 0.001; white uniform, β = 0.200, p = 0.033; nurse image, β = -0.145, p = 0.043. | r range = 0.168–0.511. Standardized β range = -0.145 to 0.489. Model R² = 0.577. |
| Cohen et al. (2004) | Nursing compared with ideal-career attributes: autonomy, busyness, hands-on work, income, job security, technology, respect, safety, caring, academic requirements, hard work. | Paired t tests: significant differences for most image-related attributes, mostly p < 0.01; college degree requirement, p = 0.030. | Effect sizes not reported. |
| Degazon et al. (2015) | Image-related attributes assessed: autonomy, income, leadership, job security, decision-making, safety, respect, appreciation, power, and work activity. | USA vs Israel perceptions of nursing: total scale and selected attributes, all p < 0.001. Within-country comparisons of nursing vs ideal-career attributes: all key discrepancies p < 0.001. | Effect sizes not reported. |
| Degazon & Shaw (2007) | Image-related domains assessed: total score, power, evaluation, and activity. | Nursing vs ideal career: total scale, Z = -5.90, p < 0.001; power, Z = -9.30, p < 0.001; evaluation, Z = -7.50, p < 0.001; activity, Z = -9.10, p < 0.001. | Effect sizes not reported. |
| Drenkard et al. (2002) | Image-related items assessed pre-post camp: leadership, teaching, executive role, programme development, demand for nurses, and scientific research. | Significant pre-post improvements across several image-related items, with standardized p-values ranging from p = 0.020 to p < 0.001. | Effect sizes not reported |
| Droes et al. (1993) | Image dimensions assessed: caring, usefulness, autonomy, scientific knowledge, and total image score. | ANCOVA intervention effects: caring, F = 20.17, p = 0.001; usefulness, F = 13.18, p = 0.001; autonomy, F = 38.85, p = 0.001; scientific knowledge, F = 34.75, p = 0.001; total image score, F = 42.72, p = 0.001. | Effect sizes not reported |
| Elmorshedy et al. (2020) | Knowledge score: median 50.0, IQR** 50.0–66.7. Nursing duties viewed as complementary to physicians: 61.0%; nursing has several specialties: 77.1%; nurse can be promoted to hospital manager: 31.1%. Perceived barriers: poor public image, 19.4% agreed/strongly agreed; mixed working environment, 35.9%; delayed marriage, 20.3%; negative effect on female social life, 64.5%. | Awareness differed by marital status, p = 0.001; education, p = 0.001; and female nurse in family, p = 0.046. Perceived barriers differed by education, p = 0.043; and female nurse in family, p = 0.032. | AOR*s: poor awareness, AOR = 1.81–2.74; perceived barriers, AOR = 0.66–1.68. |
| Joung & Shin (2018) | Q-methodology identified three nurse-image types. Factor eigenvalues: 7.15, 2.56, and 2.54; cumulative variance = 40.79%. Common positive consensus items included “valuable and worthwhile job” (z = 1.30) and “faithful/sincere/earnest” (z = 1.10). Common negative consensus items included “scientific knowledge” (z = -0.26), “leadership” (z = -0.71), and “misuses power/authority” (z = -2.00). | No inferential p-value testing was reported for image differences; Q-factor correlations ranged from r = 0.393 to r = 0.421. | Q-methodology factor statistics reported; effect size not applicable. |
| Kohler & Edwards (1990) | Nursing education/image: professional = 91%; knowledge required = 88%; college education required = 78%; money required = 70%; technical = 70%; difficult = 66%; not very long education = 32%; affordable = 18%. Work attributes: stimulating/challenging = 80%; unpleasant tasks = 82%; always find jobs = 46%; can choose days/hours = 19%. Status/pay: equal to physicians ≈ 29%; low-status occupation ≈ 29%; same as secretaries ≈ 47%; lawyers higher status ≈ 85%; nurses not well paid ≈ 50%; nurses make more than teachers ≈ 74%. | No inferential statistical comparison was reported. | Descriptive percentages only; effect size not applicable. |
| Luchesi et al. (2009) | Nursing ranked 8th of 14 professions for social status/reputation, mean rank = 7.7. Nursing was classified as masculine by 2/46 students, feminine by 16/46, and suitable for both genders by 28/46. More than 35 students (76%) described nursing care received as competent, sympathetic, and considerate. | No inferential statistical comparison was reported. | Descriptive frequencies/rankings only; effect size not applicable. |
| Marriner-Tomey et al. (1990) | Nursing mean scores were highest for going to college (M = 4.3), working hard (M = 4.2), caring for people (M = 4.2), and always having a job (M = 4.0). Lower nursing scores were reported for power (M = 3.0), making decisions (M = 3.2), safety (M = 3.3), and leadership (M = 3.3). | Paired comparisons between ideal-career and nursing attributes were reported using t values. Significant discrepancies were reported for multiple image-related attributes, including job security, caring, appreciation, knowledge, hard work, pay, hands-on work, safety, leadership, decision-making, busyness, respect, and technology. Reported t values ranged from t= -41.24 to 18.94 for key significant items. Exact p-values were not reported. | Effect sizes not reported. |
| Matutina (2012) | Mean pre-post nursing perception changes were: control = 0.123 ± 0.196; MicroCareerBursts = 0.220 ± 0.729; DNP = 0.142 ± 0.292; MicroCareerBursts + DNP = 0.192 ± 0.431. In the MicroCareerBursts group, selected nursing image items improved descriptively, including “use my brain a lot” (3.9 to 4.3), “am a leader” (3.7 to 4.0), “make decisions” (3.4 to 3.8), and “be powerful” (3.4 to 3.8). | Differences between ideal-career and nursing change scores were not statistically significant: control p = 0.493; MicroCareerBursts p = 0.502; DNP p = 0.289; MicroCareerBursts + DNP p = 0.303. MicroCareerBursts pre-post item comparisons were also not statistically significant, p = 0.130 to p = 1.000. | Effect sizes not reported. |
| May et al. (1991) | Nursing was rated highest for working hard (M = 4.34), being very busy (M = 4.28), caring for people (M = 4.27), using the brain (M = 4.26), and working with hands (M = 4.22). Lowest nursing ratings were for making a lot of money (M = 2.90), being powerful (M = 3.02), safety (M = 3.39), making decisions (M = 3.47), and leadership (M = 3.50). | Ideal-career versus nursing comparisons were significant for 12 image-related attributes, all p < 0.001. Largest t values were for making decisions, t = 22.42; safety, t = 21.08; money, t = 18.96; respect, t = 18.76; appreciation, t = 18.23; hands-on work, t = -17.44; and technology, t = -14.07. | Effect sizes not reported. |
| Maymoun & Sohail (2020) | Attitude and knowledge about nursing were assessed as image-related constructs among Emirati female students. | Structural model: R² = 0.473. Attitude was significant, β = 0.694, p < 0.001. Knowledge was not statistically significant, β = 0.027. Attitude × knowledge interaction was significant, β = 0.162, p < 0.01. | Standardized β values reported; model R² = 0.473. |
| Palazzo & Erickson (2022) | Mean scale scores: attitude M = 2.83 ± 0.61; subjective norm M = 3.05 ± 0.80; perceived behavioural control M = 2.21 ± 0.78; behavioural intention M = 3.65 ± 1.05; past behaviour M = 4.29 ± 1.03. Lower scores indicated more positive perceptions. | Gender identity was significantly associated with all image-related TPB scales: attitude F(5,814) = 22.22, p < 0.001; subjective norm F(5,814) = 15.76, p < 0.001; perceived behavioural control F(5,814) = 8.74, p < 0.001; behavioural intention F(5,814) = 14.75, p < 0.001; past behaviour F(5,814) = 11.11, p < 0.001; total scale F(5,814) = 23.35, p < 0.001. Age was not associated with scale scores, p = 0.210 to p = 0.860. | Effect sizes not reported. |
| Öncü et al. (2022) | Nursing image score: high school students M = 143.37 ± 13.90; nursing students M = 148.16 ± 11.79. Media portrayals included compassionate (48.6%), tiring (43.7%), caring (42.5%), educational (37.4%), dynamic (31.2%), angry (30.0%), sexy (29.5%), unpleasant (28.3%), and cruel (23.8%). | Nursing image total scores were higher among students who wanted to practise nursing in future, p < 0.01. Job satisfaction increased as nursing image perception improved, r = 0.385, p < 0.01. Logistic regression models were significant: high school model χ² = 105.275, p < 0.001, Nagelkerke R² = 0.177; nursing student model χ² = 89.929, p < 0.001, Nagelkerke R² = 0.335. | r = 0.385 for nursing image and job satisfaction. Regression estimates reported as Nagelkerke R² = 0.177 and 0.335. |
| Rambur et al. (2011) | Women: largest nursing-versus-ideal discrepancies were respect difference = 0.85, decision-making = 0.68, safety = 0.73, busyness = -0.77, hands-on work = -0.87, technology = -0.73. Men: largest discrepancies were respect = 0.59, college degree = 0.71, decision-making = 1.02, money = 0.93, busyness = -0.64. | Significant nursing-versus-ideal discrepancies were reported using Bonferroni-adjusted thresholds. Women: p < 0.001 for all listed discrepancies. Men: respect and busyness p < 0.001; college degree, decision-making, and money p < 0.001. | Effect sizes not reported. |
| Reiskin & Haussler (1994) | Students most often perceived nurses as caring, working with their hands, working hard, and being busy. They were least likely to see nurses as leaders, decision-makers, powerful, or highly paid. Factor reliability: ideal career α = 0.85; nursing career α = 0.89. | Significant ideal-versus-nursing differences were reported for all items, p < 0.01. Ethnic/racial group differences were significant for “nurses make a lot of money,” F = 3.04, p = 0.030, and for the evaluation factor, F = 2.72, p = 0.047. Selected gender differences were also reported, including caring, t = 3.01, p = 0.003. | Effect sizes not reported. |
| Rossiter et al. (1998) | Nursing ranked sixth overall among career preferences; 15/157 students (9.6%) selected nursing. Attitude items: fulfilling to see patients get better = 87.4%; enjoy caring/being with people = 68.1%; nursing is challenging = 52.5%; nursing is interesting = 33.8%; comfortable being a nurse = 28.8%. | Attitude differences across NESB groups were significant for comfort with being a nurse, χ² p < 0.001; nursing as interesting, χ² p < 0.001; and enjoying caring/being with people, χ² p = 0.002. Overall MANCOVA for knowledge, attitude, and intention by NESB group was significant, Wilks’ Λ = 0.802, df = 9,353, p < 0.001. Attitude correlated with intention, r = 0.47, p < 0.001. | r = 0.47 for attitude-intention association. Other effect sizes not reported |
| Rossiter et al. (1999) | Knowledge scale M = 3.68 ± 0.45; attitude scale M = 3.17 ± 0.48; intention scale M = 2.64 ± 0.58. Descriptive image items: caring for patients = 93%; skill/knowledge needed = 91%; nurses assist doctors = 86%; nursing is challenging = 67%; nursing is interesting = 18%; comfortable with being a nurse = 15%; overseas travel opportunities = 8%. | Gender differences in knowledge, attitude, and intention were not statistically significant: p = 0.974, p = 0.054, and p = 0.228. Nursing exposure group MANOVA was significant, Wilks’ Λ = 0.970, p = 0.025. Exposure-group differences were significant for attitude, p = 0.013, and intention, p = 0.008, but not knowledge, p = 0.294. Correlations: knowledge-attitude r = 0.35, p < 0.001; knowledge-intention r = 0.12, p = 0.022; attitude-intention r = 0.53, p < 0.001. | r = 0.12–0.53 for scale correlations. Other effect sizes not reported |
| Rubbi et al. (2019) | NAQ total score was higher among students who attended orientation: Group 1 M = 100.39 ± 8.70; Group 2 M = 108.40 ± 9.26. Subscale means were also higher in Group 2: professional role 36.68 vs 33.79; professional values 22.44 vs 20.98; stereotypes 21.61 vs 19.78; professionalism 16.32 vs 15.32; characteristics of nurses/nursing 11.35 vs 10.53. | Orientation-group differences were significant for total NAQ and all subscales, all p < 0.001. Total NAQ: t = -7.752, p < 0.001. Professional role: t = -6.761, p < 0.001; professional values: t = -4.422, p < 0.001; stereotypes: t = -6.395, p < 0.001; professionalism: t = -4.100, p < 0.001; characteristics: t = -3.974, p < 0.001. | Effect sizes not reported. |
| Tang et al. (1999) | Positive image items: nurses help people = 97.6%; care for sick/wounded/frail = 95.8%; work with hands = 90.1%; work very hard = 90.0%; appreciated = 85.6%; respected = 78.6%; varied settings = 75.6%; university degree = 72.0%. Less positive items: leadership = 35.7%; powerful = 36.5%; make a lot of money = 38.5%; decision-making = 41.7%. | Gender difference was significant only for perception that nurses work in a safe environment, t = 2.01, p = 0.045. ANOVA showed language-group differences across five of six nursing-image factors: influence, job nature, knowledge, social recognition, and job context; post-hoc differences were reported at p < 0.05. No significant language-group difference was reported for job opportunities. | Effect sizes not reported. |
| Mohsen et al. (2022) | Public image of nursing M = 4.38 ± 0.81. Students with nurse exposure had higher public image scores than those without exposure: M = 4.46 ± 0.77 vs M = 4.08 ± 0.85. Public image also differed by paternal education: elementary M = 1.40 ± 0.23; junior high M = 2.23 ± 0.54; high school M = 2.58 ± 0.79; academic M = 2.31 ± 0.57. | Public image differed by paternal education, F = 3.54, p < 0.05. Public image differed by past exposure to nurses, t = 2.08, p < 0.05. Public image correlated with intention to choose a healthcare profession, r = 0.23, p < 0.01. Regression: public image of nursing was significant, B = 0.67, β = 0.35, t = 4.13, p < 0.001; model R² = 0.21. | r = 0.23; standardized β = 0.35; R² = 0.21. |

Note. *AOR = adjusted odds ratio; **IQR = interquartile range; Exact p-values were retained where reported; p-values reported as .000, .0001, or < .0001 in the original studies were standardized as p < 0.001. Effect sizes were reported where available.

**Table S8** *Quality Assessment Results Mixed Methods Appraisal Tool*

References:

Cho Tang, K., C. Duffield, X. C. Chen , et al. 1999 . “ Nursing as a Career Choice: Perceptions of Students Speaking Arabic, Serbo-Croatian, Spanish, Turkish or Vietnamese at Home .” *Australian Health Review* 22, no. 1: 07–121. <https://doi.org/10.1071/ah990107>.

Degazon, C. E., and H. K. Shaw. 2007. “Urban High School Students' Perceptions of Nursing as a Career Choice.” *Journal of National Black Nurses' Association* 18, no. 1: 8–13.

Luchesi, L. B., I. A. C. Mendes, G. T. Shiniyashiki, and M. L. D. A Costa Junior. 2009. “Elaboração de Instrumento Para Análise da Imagem do Enfermeiro Frente a Alunos do Ensino Médio.” *Revista da Escola de Enfermagem da USP* 43, no. 2: 272–278. <https://doi.org/10.1590/S0080-62342009000200003>.

Mohsen, A., I. Bluvstein, R. Wilf Miron, and I. Kagan. 2022. “Public Image of the Profession Is Associated With the Choice of Nursing Career Among Arab High School Students: A Cross-Sectional Study.” *Journal of Nursing Management* 30, no. 1: 310–317. <https://doi.org/10.1111/jonm.13454>.

Palazzo, S. J., and M. S. Erickson. 2022. “Male High School Students' Perceptions of Nursing as a Professional Career Choice: Nursing Pathway Project.” *Nursing Education Perspectives* 43, no. 2: 96–102. <https://doi.org/10.1097/01.Nep.0000000000000897>.

Rambur, B., M. V. Palumbo, B. McIntosh, J. Cohen, and S. Naud. 2011. “EXTRA Young Adults' Perception of an Ideal Career.” *Nursing Management* 42, no. 4: 19–24. <https://doi.org/10.1097/01.Numa.0000395265.82233.10>.

Rossiter, J. C., J. Bidewell, and P. T. Chan. 1998. “Non-English Speaking Background High School Students' Attitudes Towards the Nursing Profession.” *Journal of Advanced Nursing* 27, no. 3: 604–613. <https://doi.org/10.1046/j.1365-2648.1998.00543.x>.
